# Drug‐Event Pairs as Indicators for the Detection of Adverse Drug Reactions during Hospitalization in Routinely Collected Electronic Data Sources

**DOI:** 10.1002/cpt.3635

**Published:** 2025-03-18

**Authors:** Anna Maria Wermund, Annette Haerdtlein, Wolfgang Fehrmann, Clara Weglage, Tobias Dreischulte, Ulrich Jaehde

**Affiliations:** ^1^ Department of Clinical Pharmacy, Institute of Pharmacy University of Bonn Bonn Germany; ^2^ Institute of General Practice and Family Medicine LMU University Hospital, LMU Munich Munich Germany

## Abstract

Adverse drug reactions (ADRs) are a common cause of morbidity and mortality in hospitalized patients. Identification of ADRs in clinical practice, surveillance and research is essential to prevent further harm. The aim of this study was to assess the likelihood of drugs contributing to clinically important inpatient adverse events, in order to provide a list of drug‐event pairs indicating ADRs in electronic health record (EHR) data, referred to as “indicators of ADRs”. We conducted a consensus process based on the RAND/UCLA Appropriateness Method for 14 ADRs. Experts were asked to rate the strength of the causal link between adverse events and potentially causative drugs on a 4‐point Likert scale. Based on the median rating, drug‐event pairs were categorized according to the likelihood of an ADR being present. Drug‐event pairs with a median rating of ≥ 3 without disagreement were defined as indicators of certain and probable ADRs. Of the 255 drug‐event pairs evaluated, 2 (1%) and 42 (16%) achieved consensus validation that they certainly and probably indicate an ADR. In addition, 137 drug‐event pairs were considered as indicators of possible (54%) and 74 drug‐event pairs were considered as indicators of unlikely (29%) ADRs. The provided set of content‐validated indicators of clinically important inpatient ADRs can be used in clinical practice (e.g., decision support), surveillance (e.g., quality indicators) and research (e.g., outcome measures). They will be implemented in EHR data from German university hospitals to determine the prevalence of ADRs, support efficient use of pharmacist resources, and develop models predicting ADRs.

**Study Highlights**

**WHAT IS THE CURRENT KNOWLEDGE ON THE TOPIC?**

Conventional methods of detecting adverse drug reactions (ADRs), such as voluntary incident reporting, retrospective chart review, and direct observation in prospective ADR surveillance, have limitations in terms of effectiveness and affordability. Increasingly, these methods are supplemented by electronic triggers, mainly consisting of a single information item. As most ADRs require more than one information item to be identified and the associated drugs differ between ADR categories, combining adverse events with potentially causative drugs could facilitate ADR detection.

**WHAT QUESTION DID THIS STUDY ADDRESS?**

The experts were asked to rate the strength of the causal link between adverse events and potentially causative drugs by answering the question, “How likely is it that the listed medication significantly contributed to the adverse event, so that you would assume an adverse drug reaction?”

**WHAT DOES THIS STUDY ADD TO OUR KNOWLEDGE?**

For 14 clinically important inpatient ADRs, content‐validated drug‐event pairs as indicators of ADRs are provided for implementation in routine electronic data sources. Categorized into 2 indicators of certain, 42 indicators of probable, 137 indicators of possible, and 74 indicators of unlikely ADRs, they inform about the likelihood of an ADR being present.

**HOW MIGHT THIS CHANGE CLINICAL PHARMACOLOGY OR TRANSLATIONAL SCIENCE?**

The content‐validated indicators can be applied in clinical practice (e.g., decision support), clinical surveillance (e.g., as quality indicators) and research (e.g., as outcome measures) to detect ADRs and to promote drug safety studies under real‐world hospital conditions.

The detection, characterization and prevention of adverse drug reactions (ADRs) remains a major challenge for the World Health Organization (WHO) and other health agencies worldwide,^1^, ^2^ as they are a common cause of morbidity and mortality across all health care settings.^3^ For the hospital setting, a prospective observational study showed that ADRs affected 15% of hospitalized patients and prolonged their hospital stay by an average of 0.25 days/patient admission episode.^4^ An ADR is defined as “a response to a medicinal product which is noxious and unintended”. According to this definition, the causal relationship between the drug and the adverse event (AE) is at least a reasonable possibility. Therefore, an ADR must be clearly distinguished from an AE, defined as “any untoward medical occurrence in a patient to whom a medicinal product is administered and which does not necessarily have a causal relationship with this treatment.” A causal relationship is therefore suspected for an ADR, but is not required for an AE.^5^

Detecting and quantifying ADRs and their causes is essential to prevent further harm, identify safety priorities, and improve the quality of care through the development of remedial action plans.^6^, ^7^, ^8^ However, conventional methods of ADR detection, such as voluntary incident reporting, retrospective chart review, and direct observation in prospective ADR surveillance, have limitations in terms of effectiveness and affordability.^8^ Increasingly, these methods have been complemented by the use of electronic trigger tools: computer‐based algorithms that automatically screen routinely collected, readily available electronic data and flag simple patterns suggestive of a past, present, or future ADR.^8^, ^9^, ^10^ Implemented in electronic health records (EHR), they can be used efficiently at the point of care to automatically screen for potential ADRs, assess the overall harm caused by medical care, and measure changes in the occurrence of potential ADRs on a large scale for clinical surveillance and research.^9^, ^11^, ^12^ Several sets of triggers have been developed, ranging from global lists of triggers for a large number of AEs^13^ to very specific lists that differ according to the specific type of AEs (e.g., ADR),^14^ clinical setting (e.g., oncology) or target patient population (e.g., pediatric, elderly).^15^, ^16^, ^17^, ^18^ Electronic trigger tools are moderately effective, time‐efficient to use, and have been shown to be less burdensome than conventional methods of ADR detection.^19^, ^20^, ^21^, ^22^ This makes them a suitable tool for the Germany‐wide POLAR_MI (POLypharmacy, drug interActions and Risks) project of the Medical Informatics Initiative Germany, which aims to detect medication‐related risks using EHR data from university hospitals, including inpatient ADRs.^23^ However, triggers of existing tools are mainly based on a single variable (e.g., only blood glucose < 50 mg/dL, only digoxin level > 2 ng/mL, only use of diphenhydramine), which are rather suitable for the detection of AEs and limit their positive predictive values regardless of the data category.^9^, ^10^, ^24^, ^25^

Given the variability in the likelihood of drugs causing specific ADRs and the differing levels of clinical importance of ADRs, the combination of clinically important and highly drug‐related AEs with potentially causative drugs can focus the detection of ADRs in EHRs on those with (at least) probable drug‐related causes, potentially enhancing the predictive performance and specificity of ADR detection.^3^, ^26^, ^27^ To achieve this goal, we aimed to assess the likelihood of specific drugs contributing to clinically important inpatient AEs, in order to provide a consensus‐based list of drug‐event pairs indicating ADRs in EHR data, hereafter referred to as “indicators of ADRs”. The drug‐event pairs were categorized according to the likelihood of an ADR being present.

## MATERIAL AND METHODS

### Study design and selection of ADRs

**Figure 1:**
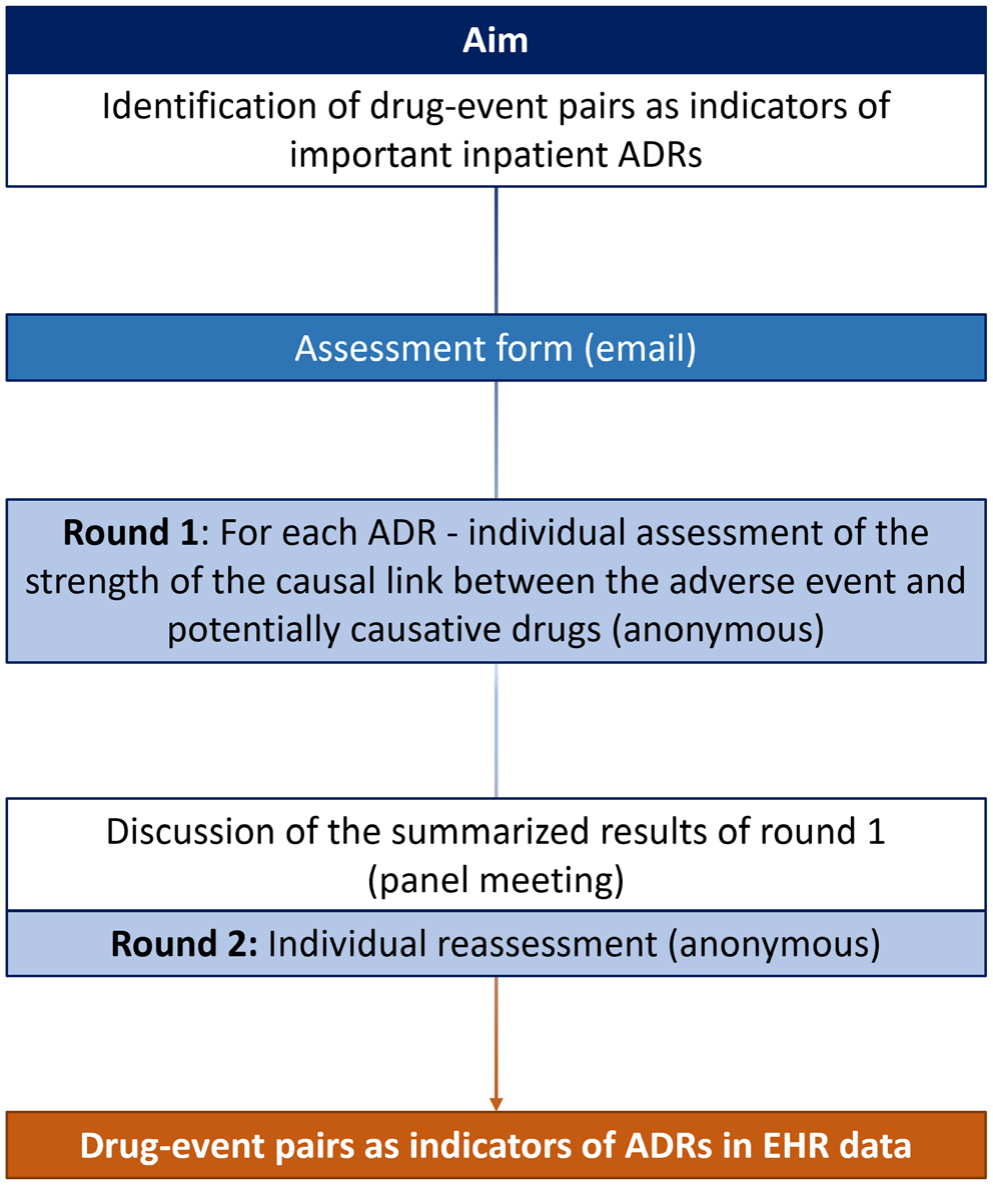
RAM consensus process (ADRs, adverse drug reactions; EHR, electronic health record).

We conducted an expert consensus process based on the RAND/UCLA Appropriateness Method (RAM), a variant of the Delphi method that combines scientific evidence and expert opinion.^28^ In consensus processes based on the RAM method, the experts rate clinical presentations in a two‐round rating process, taking into account the available evidence. In the first round, experts rate each clinical presentation independently. In the second round, after a panel meeting to review and discuss first‐round ratings and revise the initial list of presentations, the experts re‐rate each clinical presentation individually. In the consensus process presented here, we followed this procedure (**Figure** **1**). The experts were asked to rate the strength of the causal link between an AE and potentially causative drugs (as individual drugs or grouped into drug classes) in order to identify drug‐event pairs indicating inpatient ADRs in EHR data.

The AEs considered were selected through a different, previous consensus process in which experts were asked to rate the clinical importance of AEs in the context of drug safety on a 4‐point Likert scale (1 = not important to 4 = very important).^26^ In this consensus process, 14 AEs had a median importance rating of 4 (=very important) and were therefore included in the consensus process presented here: rhabdomyolysis, acute kidney injury (AKI), hypoglycemia, liver damage, anaphylaxis, delirium, hyperkalemia, serotonin syndrome, bleeding outside the gastrointestinal tract (GIT), agranulocytosis and neutropenia, Stevens‐Johnson syndrome (SJS) and toxic epidermal necrolysis (TEN), ventricular tachycardia, bleeding of the upper GIT and hyponatremia. The AE “ventricular tachycardia” was changed to “torsade de pointes (TdP) tachycardia”, as this specific type of tachycardia is more commonly caused by drugs.^29^

The Ethics Committee of the Medical Faculty of the University of Bonn, Germany (AZ 2021–502) exempted the study from institutional review board review.

### Selection of experts

We attempted to re‐recruit all the experts who participated in the previous consensus process by which the AEs considered here were selected.^26^ Aiming for a minimum of nine experts, we sought a new expert if one was no longer available. In general, we recruited pharmacists and physicians with an academic interest or clinical experience in the detection or management of inpatient ADRs, aiming for a balanced distribution of the two professions and of self‐reported (predominant) professional activity as scientist or clinician. We used the mailing list of the POLAR_MI project and asked involved experts to nominate further experts for recruiting.

### Literature search on potentially causative drugs

A structured literature search was conducted to generate comprehensive lists of potentially causative drugs for each ADR. A standard operating procedure was developed, defining the search strategy, *Medical Subject Headings*, keywords, inclusion criteria, and extraction method (**Supplement** **1**). We searched MEDLINE® for articles published between 2010 and 2021 or 2000 and 2021, depending on the amount of literature found in the 2010–2021 period. The search strategy consisted of one part describing the specific AE, combined with another part establishing the drug association. As reviews have been published for most AEs, we focused on this type of article and also included a reference book by Anne Lee.^30^ We did not include information from summary of product characteristics (SmPCs) as we wanted to rely on scientific evidence rather than regulatory information, which is often insufficient to assess the strength of the causal link and varies between manufacturers. As the literature on liver damage and TdP tachycardia mainly refers to the databases Livertox® and CredibleMeds®, the potential causative drugs for these AEs were extracted from these databases.^29^, ^31^

### Extraction and grouping of potentially causative drugs

From selected publications, we extracted all drugs and drug classes for which a causal relationship with the AE was described and grouped them into superordinate drug classes, as it was not feasible to assess all individual drugs. If a specific drug class was mentioned in the literature, the experts were asked to consider the whole group. If only certain substances from a drug class were mentioned, but not the class itself, we investigated whether there was a group effect. If there was sufficient evidence, the individual drugs were combined into a drug class to be considered as a whole group. If there was insufficient evidence but the strength of the causal relationship was considered to be similar, the drugs were grouped together, and the experts were asked to assess only these certain drugs (e.g., certain antiepileptics: phenytoin, carbamazepine). All drugs that could not be assigned to a drug class were grouped as “miscellaneous drugs” In order to reduce the number of drugs in the miscellaneous groups and the number of other drug classes to be rated, drugs and drug classes with less evidence of causing the AE were excluded. Evidence was considered weak if only one reference was found in the literature search and no further evidence could be identified from other sources (e.g., summary of product characteristics). For the AEs liver damage and TdP tachycardia, the categories from LiverTox® and CredibleMeds® were used as drug classes, respectively (e.g., 1 drug from category A according to LiverTox®).^29^, ^31^

### RAM procedure

#### Design of the assessment form

The assessment form was a Microsoft Excel™ document with an instruction sheet and separate sheets for each AE. A sample of the round one assessment form is provided in **Supplement** **2**.^32^, ^33^, ^34^, ^35^, ^36^ The sheets listed the potentially causative drug classes, with specifications of individual drugs, where only certain drugs within each class were to be considered. In addition to the columns for ratings and comments, there was a separate column for the evidence report that the experts were asked to consider in their assessment. The evidence report included a description of the mechanism of action causing the ADR and the empirical evidence summarized from the publications from which the drugs were extracted.

#### Assessment criterion and pre‐specifications

**Figure 2:**
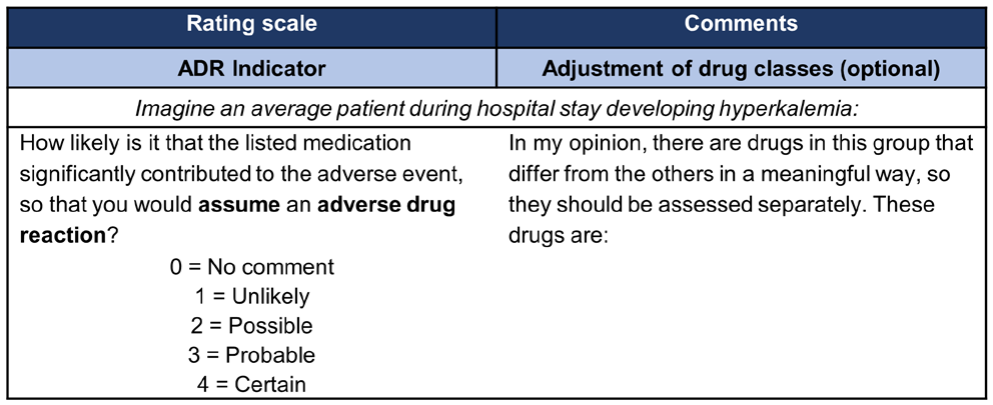
Assessment criterion and rating scale using hyperkalemia as an example (ADR, adverse drug reaction).

The experts were asked to rate the strength of the causal link between the AE and potentially causative drugs (drug‐event pairs) by answering the question “How likely is it that the listed medication significantly contributed to the adverse event, so that you would assume an adverse drug reaction?” in relation to an average patient and assuming the drug exposure at the time of the AE. It was also pre‐specified that all AEs should be assessed in relation to ADRs that occur acutely during hospitalization, require treatment and are not due to underuse or discontinuation of a drug. The 4‐point Likert scale was based on the causality terms from the WHO‐UMC system for standardized causality assessment.^37^ The experts were given the opportunity to abstain (0 = no comment) if they were unable to assess a drug class despite the evidence provided. In addition, they could make comments on the composition of the drug classes (**Figure** **2**).

To determine the threshold for the presence of an ADR, we also asked about the likelihood of an ADR being present if two drugs rated as possible or two drugs rated as probable were listed together with the AE in the EHR data (drug combination‐event pairs).

The drugs and drug classes that were excluded due to insufficient evidence were listed below the drug classes to be rated, so that the experts could indicate whether they wanted to include one of these drugs in the assessment form. In a free text field, experts could add other drugs that were not included in the assessment form based on their own clinical experience.

#### Analysis of the ratings

Drug‐event pairs with a median rating of 4 and 3 without disagreement were predefined as indicators of certain and probable ADRs, respectively. Disagreement was predefined to be present if at least 30% of expert ratings were < 3 (for drug‐event pairs with a median of ≥ 3), or 3 or higher (for drug‐event pairs with a median of < 3). Drug‐event pairs with a median rating of ≥ 3 with disagreement or with a median rating of 2 or 2.5 with and without disagreement were considered indicators of possible ADRs. All drug‐event pairs with a median rating of < 2 were predefined as indicators of unlikely ADRs.

#### Rating rounds

The assessment form was sent to the experts by email. Six weeks after the first round, an expert meeting took place, moderated by UJ. For each drug‐event pair, the first‐round ratings were summarized and fed back to the experts. To facilitate discussion, each ADR was discussed separately. The focus of the discussion was on drug‐event pairs with disagreement and on drug classes where experts recommended splitting. Discussions were also held for all low‐evidence drugs recommended for inclusion. The same applied to all other substances added by the experts. After discussion of all potentially causative drugs for an ADR, the panelists directly placed their second‐round ratings.

### Pre‐test and optimization

The assessment form was pre‐tested and optimized in two steps. Each step involved a pharmacist and a physician (who were not part of the research team or the expert panel). In the first step, the draft of the assessment form was presented to the experts. In the second step, another two experts were presented with a revised assessment form. In both steps, feedback was obtained through semi‐structured interviews (interview guide: **Supplement** **3**), focusing on the formulation and definition of the assessment criterion, the comprehensibility and completeness of the drug classes, the instructions, the evidence report, and the design of the assessment form. Implementing modifications based on the second step of feedback yielded the final assessment form.

## RESULTS

### Expert panel

The expert panel consisted of five physicians and five pharmacists from nine German university hospitals. Eight experts from the preliminary consensus process also participated in the consensus process presented here. As shown in **Table** **1**, all experts had additional research or clinical qualifications.

**Table 1:** Composition of the expert panel

Characteristics	Physicians (*n* = 5)	Pharmacists (*n* = 5)
*Academic background*		
Additional qualification (habilitation/doctorate and/or clinical specialist qualification)	5 (100%)	5 (100%)
*Main field of professional activity*		
Scientific research	4 (80%)	1 (20%)
Clinical practice	0 (0%)	1 (20%)
Both	1 (20%)	3 (60%)

### Literature search and design of the assessment form

Depending on the ADR, the literature search yielded between 5 and 80 publications, used as the basis for each assessment sheet (details: **Supplement** **1**). In total, the extraction and grouping of potentially causative drugs resulted in 279 superordinate drug classes after the pre‐test. Due to insufficient evidence, 46 drug classes and 105 drugs from the miscellaneous groups were excluded, resulting in 233 drug classes to be rated in combination with certain AEs in round one. The excluded drugs and drug classes are listed in **Supplement** **4**. As the literature search for rhabdomyolysis identified the drug‐induced rhabdomyolysis atlas (DIRA), a database that contains a classification scheme for drugs causing rhabdomyolysis based on drug labeling information, the risk categories of this database were included as drug classes.^38^ For delirium, the ACB score by Kiesel et al. was used to map the anticholinergic drugs.^39^

### Rating process and findings

The round one assessment form was emailed to panelists in February 2022, and the panel of experts met on 30 March 2022. All 10 experts returned a fully completed round one assessment form, while nine experts took part in the moderated expert discussion and returned a fully completed round two assessment form. All drug‐event pairs rated, as well as detailed first‐round and second‐round ratings, are provided in **Supplement** **5**.

#### Drug‐event pairs as indicators of ADRs

**Figure 3:**
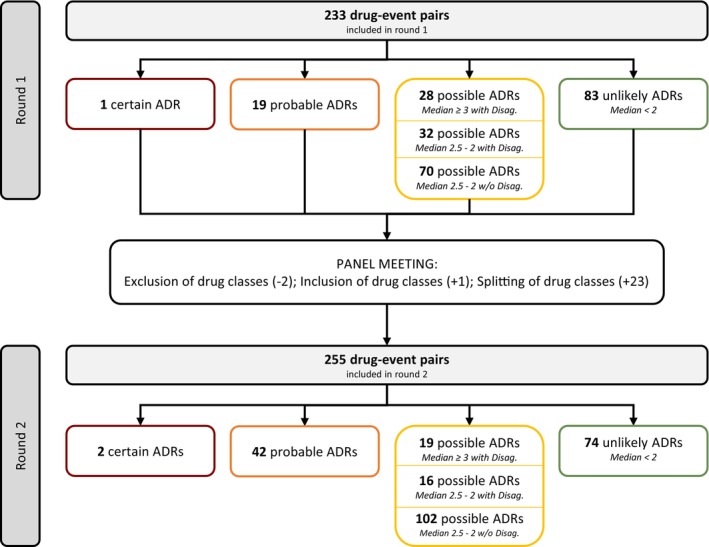
Flowchart showing the results of the rating process of the drug‐event pairs considered in the consensus process (ADRs, adverse drug reactions; Disag., disagreement; w/o, without).

In round one, of the 233 drug‐event pairs rated, disagreement was present for 60 drug‐event pairs (26%). This resulted in 1 drug‐event pair considered as an indicator of a certain ADR (0.4%), 19 drug‐event pairs considered as indicators of probable ADRs (8%), 130 drug‐event pairs considered as indicators of possible ADRs (56%) and 83 drug‐event pairs considered as indicators of unlikely ADRs (36%) after round one (see **Figure** **3**).

Of the excluded drugs due to low evidence, 17 were considered by the experts in round one and were therefore discussed during the panel meeting. It was decided to add the drug class “fibrinolytics” for the AE “bleeding outside the GIT”. During the discussion, the experts also decided to split 21 drug classes into 44 drug classes (e.g., “contrast media” was split into “iodinated contrast media” and “other contrast media” for the AE “anaphylaxis”), to change the definition of four drug classes (e.g., for “AKI”, the class “polymyxins” was changed to “polymyxins (i.v.)”) and to exclude two drug classes because they cause a different ADR that can lead to the ADR being assessed (details of the adjustments are provided in **Supplement** **6**). Therefore, 255 drug‐event pairs were rated in round two. The second assessment round resolved first‐round disagreements for 24 drug‐event pairs without any adjustment of the drug class (14 of which were now indicators of probable and 9 were indicators of possible ADRs; one pair was excluded). Disagreement could also be resolved for 8 drug‐event pairs through splitting into 17 drug‐event pairs (7 of which were now indicators of probable and 10 were indicators of possible ADRs). However, disagreement remained for 28 pre‐existing drug‐event pairs and 7 new drug‐event pairs. Finally, this resulted in 2 drug‐event pairs considered as indicators of certain ADRs (1%), 42 drug‐event pairs considered as indicators of probable ADRs (16%), 137 drug‐event pairs considered as indicators of possible ADRs (54%) and 74 drug‐event pairs considered as indicators of unlikely ADRs (29%) after round two (see **Figure** **3**). The distribution of ADR categories after round two is shown in **Table** **2**.

**Table 2:** Distribution of drug‐event pairs across the ADR categories after round two

Adverse drug reaction	N Drug classes	N certain (%)	N probable (%)	N possible (%)	N unlikely (%)
Rhabdomyolysis	22	0 (0.0%)	2 (9.1%)	10 (45.5%)	10 (45.5%)
Acute kidney injury	32	0 (0.0%)	7 (21.9%)	18 (56.3%)	7 (21.9%)
Hypoglycemia	20	2 (10.0%)	0 (0.0%)	5 (25.0%)	13 (65.0%)
Liver damage	6	0 (0.0%)	1 (16.7%)	3 (50.0%)	2 (33.3%)
Anaphylaxis	27	0 (0.0%)	4 (14.8%)	19 (70.4%)	4 (14.8%)
Delirium	17	0 (0.0%)	2 (11.8%)	8 (47.1%)	7 (41.2%)
Bleeding outside the GIT	17	0 (0.0%)	7 (41.2%)	6 (35.3%)	4 (23.5%)
Hyperkalemia	16	0 (0.0%)	1 (6.3%)	13 (81.3%)	2 (12.5%)
Agranulocytosis/Neutropenia	33	0 (0.0%)	4 (12.1%)	12 (36.4%)	17 (51.5%)
TdP tachycardia	8	0 (0.0%)	3 (37.5%)	5 (62.5%)	0 (0.0%)
Bleeding of the upper GIT	10	0 (0.0%)	4 (40.0%)	4 (40.0%)	2 (20.0%)
Serotonin syndrome	15	0 (0.0%)	3 (20.0%)	11 (73.3%)	1 (6.7%)
SJS/TEN	14	0 (0.0%)	0 (0.0%)	14 (100.0%)	0 (0.0%)
Hyponatremia	18	0 (0.0%)	4 (22.2%)	9 (50.0%)	5 (27.8%)

GIT, Gastrointestinal tract; N, Number; SJS/TEN, Stevens‐Johnson syndrome and toxic epidermal necrolysis; TdP, Torsade de pointes.

#### Drug combination‐event pairs as indicators of ADRs

Regarding the combinations of two drugs rated as possible and two drugs rated as probable, for hyperkalemia, delirium and serotonin syndrome there was agreement after round two that the concomitant use of two drugs rated as possible together with the presence of the related AE (drug combination‐event pair) is probable indicating an ADR. The concomitant use of two drugs rated as “3 = probable” never had a median rating of “4 = certain”. This resulted in 3 additional indicators of probable ADRs, consisting of two drugs with a possible rating and the AE. A list of all indicators of certain and probable ADRs can be found in in **Table** **3**.

**Table 3:** Ready‐to‐use list of all indicators of certain and probable ADRs

Adverse event	Drug class
*Rhabdomyolysis*	
Rhabdomyolysis	Statins
Rhabdomyolysis	Trabectedin
*Acute kidney injury*	
Acute kidney injury	NSAIDs
Acute kidney injury	Aminoglycosides
Acute kidney injury	Vancomycin
Acute kidney injury	Methotrexate/Cisplatin/Ifosfamide
Acute kidney injury	Certain antivirals (Nucleoside analogues, Cidofovir, Foscarnet)
Acute kidney injury	Contrast agents (i.v.)
Acute kidney injury	Calcineurin inhibitors
*Hypoglycemia*	
Hypoglycemia	Insulin
Hypoglycemia	Sulfonylureas
*Liver damage*	
Liver damage	1 drug from category A according to LiverTox®
*Anaphylaxis*	
Anaphylaxis	Beta‐lactams
Anaphylaxis	Vancomycin
Anaphylaxis	Iodinated contrast media
Anaphylaxis	Biologicals with immunological target
*Delirium*	
Delirium	Total ACB score: ≥ 3 points
Delirium	Narcotics
	Two drugs rated as possible: Total ACB Score: ≥ 1 point; SSRI (excl. Paroxetine), Anticonvulsants (excl. Pheno‐barbitals and Carbamazepine), Dopamine agonists, GABA‐receptor agonists, Opiates, Miscellaneous drugs
*Bleeding outside the GIT*	
Bleeding outside the GIT	ASA
Bleeding outside the GIT	Heparins
Bleeding outside the GIT	Vitamin K antagonists
Bleeding outside the GIT	Direct oral anticoagulants
Bleeding outside the GIT	Certain other anticoagulants (Fondaparinux, Argatroban, Bivalirudin)
Bleeding outside the GIT	Other antiplatelet drugs (excl. ASA, Dipyridamole, Cilostazol)
Bleeding outside the GIT	Fibrinolytics
*Hyperkalemia*	
Hyperkalemia	Agents containing a high amount of potassium
	Two drugs rated as possible: ACE inhibitors, ARBs, Direct renin inhibitors, Aldosterone antagonists, ENaC blockers, NSAIDs, Heparin and derivatives, Tacrolimus, Other calcineurin inhibitors, Pentamidine, Cotrimoxazole, Miscellaneous drugs, Suxamethonium
*Agranulocytosis/Neutropenia*	
Agranulocytosis/Neutropenia	Cytotoxic anticancer drugs
Agranulocytosis/Neutropenia	Clozapine
Agranulocytosis/Neutropenia	Pyrazolones
Agranulocytosis/Neutropenia	Mycophenolate mofetil/Azathioprine
*Torsade de pointes tachycardia (CredibleMeds® categories)* a	
Torsade de pointes tachycardia	1 drug with known risk
*Bleeding of the upper GIT*	
Bleeding of the upper GIT	NSAIDs (non‐selective COX inhibitors)
Bleeding of the upper GIT	Direct oral anticoagulants
Bleeding of the upper GIT	Vitamin K antagonists
Bleeding of the upper GIT	Antiplatelet drugs
*Serotonin syndrome*	
Serotonin syndrome	SSRI
Serotonin syndrome	SSNRI
Serotonin syndrome	MAO inhibitors
	Two drugs rated as possible: Clomipramine, Imipramine and other TCA, Tetracyclic antidepressants, 5‐HT2A antagonists, Certain atypical antipsychotics, Triptans, Certain antiemetics, Certain opioids, Certain antibiotics with MAO‐inhibiting activity, Amphetamines and derivatives, Miscellaneous drugs
*Stevens‐Johnson syndrome (SJS) and toxic epidermal necrolysis (TEN)*	
No drug‐event pair had a median rating of ≥ 3 without disagreement	
*Hyponatremia*	
Hyponatremia	SSNRI/SSRI
Hyponatremia	Thiazides
Hyponatremia	Other diuretics
Hyponatremia	Vasopressin und analogues

5‐HT2A, 5‐hydroxytryptamine 2A receptor; ACB score, Anticholinergic burden score by Kiesel et al.; ACE, Angiotensin‐converting enzyme; ARBs, Angiotensin receptor blockers; ASA, Acetylsalicylic acid; COX, Cyclooxygenase; ENaC, Epithelial sodium channel; Excl., Exclusive; GABA, Gamma‐aminobutyric acid; GIT, Gastrointestinal tract; MAO, Monoamine oxidase; NSAIDs, Non‐steroidal anti‐inflammatory drugs; SSNRI, Selective serotonin and norepinephrine reuptake inhibitors; SSRI, Selective serotonin reuptake inhibitors; TCA, Tricyclic antidepressants.

^a^ Please note: Initially, “1 drug with known risk”, “2 drugs taken simultaneously with known risk” and “1 drug with known risk + 1 drug with possible risk taken simultaneously” had a median rating of 3 without disagreement and were therefore categorized as indicators of probable ADRs. As “1 drug with known risk” is part of all these combinations, we collapsed these three indicators into one indicator for the ready‐to‐use list.

## DISCUSSION

### Summary of findings

This study provides a set of content‐validated drug‐event pairs as indicators of clinically important inpatient ADRs in EHR data. Of the 255 drug‐event pairs evaluated, 2 (1%) and 42 (16%) achieved consensus validation that they certainly and probably indicate an ADR, respectively. In contrast, more than half of the evaluated drug‐event pairs were confirmed as only possibly indicating an ADR. This reflects the complex interplay of triggering factors (e.g., drugs) and confounding factors (e.g., underlying disease), leading to uncertainty in assessing whether an ADR is present or not. This remains a challenge even in prospective observational studies, where confounding factors can be better addressed. In the ADRED study, which investigated ADR cases in four emergency departments in Germany, 87.6% of suspected drugs were classified as possibly causal and only 12.4% as probably or definitely causal.^40^ This shows that a simple yes or no decision regarding the presence of ADRs does not capture their nature, supporting our approach to categorize the drug‐event pairs into different probability levels instead of a binary categorization (yes/no). With our approach, drugs with a lower probability of causing a specific ADR could be distinguished from those with a higher probability.

### Concomitant use of drugs and the probability of an ADR

To account for the complex relationship between ADR‐triggering factors, we also asked whether the combination of two drugs is more likely to indicate an ADR than one drug alone. For all AEs, the concomitant use of two drugs, both with a rating of “3 = probable”, never had a median rating of “4 = certain”. This highlights again the uncertainty in ADR assessment, as even when two drugs with a strong causal relationship to the AE were used together, the experts were not certain of an ADR.

For hyperkalemia, delirium, and serotonin syndrome, there was consensus that the concomitant use of two drugs classified as possibly indicating an ADR is probably indicating an ADR, highlighting their stronger multidrug etiology compared to other ADRs such as anaphylaxis and SJS/TEN, being hypersensitivity reactions where usually one causative drug is sufficient to trigger the ADR. In general, all drugs that cause the same ADR can be expected to have a potentiating effect. However, for some ADRs, this is intrinsic to their etiology. Regarding hyperkalemia, it has been reported that the risk increases with each potassium‐altering drug taken.^32^, ^33^, ^34^ The incidence of hyperkalemia is low in large controlled clinical trials of angiotensin‐converting enzyme inhibitors, angiotensin receptor blockers, and aldosterone antagonists, whereas a higher incidence is observed in clinical practice due to the co‐administration of these potassium‐altering drugs.^32^, ^33^, ^34^ There is also a common perception of a multidrug etiology for serotonin syndrome, as serotonin toxicity usually occurs with the co‐administration of two or more serotonergic drugs, especially if they increase the serotonin level in the synaptic cleft in different ways.^41^, ^42^, ^43^ Similarly, the likelihood of developing delirium increases with the number of predisposing factors, which often include more than one drug.^44^, ^45^

### Comparison with other trigger tools

There are already two trigger lists consisting of causative drugs for specific ADRs. However, both were developed to detect ADR‐related hospital admissions in the elderly and do not include a probability categorization of the drugs listed.^17^, ^46^ Delirium, AKI, bleeding (not separated into GIT and non‐GIT bleeding), hypoglycemia, hyperkalemia, and hyponatremia are also covered by these trigger lists. Noorda et al. mostly considered 2 to 4 drug classes, while Thevelin et al. included between 4 and 18 drug classes per event.^17^, ^46^ With between 6 and 33 drug classes per event, we listed even more, but the categorization resulted in a more manageable number of one to seven indicators of at least probable ADRs per event. Almost all drug classes on the trigger lists cited were part of our consensus process, underlining the comprehensiveness of our list of potentially causative drugs. Only angiotensin receptor blockers for hyponatremia and monoamine oxidase inhibitors for hypoglycemia were not included in our consensus process.^17^, ^46^ The drug classes considered in our consensus process, but not on the trigger lists cited, mostly had a median rating of one, emphasizing that these drugs are unlikely to cause the ADR and are thus not required on a trigger list for this ADR. With the exception of fluoroquinolones and digoxin for delirium, all drug classes on the cited trigger lists had a median rating of ≥2, demonstrating that our consensus process was able to discriminate well between drug‐event pairs unlikely to indicate an ADR and those indicating at least possible ADRs.

### Strengths and limitations

A strength of the developed indicators is that they link clinically important AEs to causative drugs, leading to ADR indicators categorized into different probability levels. Pre‐testing the assessment form in two steps minimized ambiguities in rating constructs and in the composition of drug classes. Any remaining misunderstandings could be clarified during the panel meeting, which also allowed for an exchange of arguments and experiences for the panelists to consider in their second‐round ratings, which is a key strength of RAM. Specific strengths of our consensus process are the heterogeneous composition of the expert panel, with diverse knowledge and experience in ADR detection, as well as the categorization of a wide range of causative drugs, as it was based on a broad literature search leading to extensive lists of potentially causative drugs. Additionally, the experts were able to indicate when a drug was missing (used for only one AE).

A limitation of our consensus process was that, for feasibility reasons, potentially causative drugs had to be grouped into superordinate and miscellaneous drug classes, partly resulting in a heterogeneous composition. Although we tried to limit this by reviewing the evidence during grouping and by pre‐testing the assessment form twice, there was still some ambiguity about the composition, especially during the first round. As the assessment form included the option of commenting if a drug class was too broad, these problems could be collected and discussed during the panel meeting. This led to the splitting of drug classes for the second rating round, resolving prior ambiguities. Our main limitation is that although the indicators developed are evidence‐based, further validation is needed by actually using them to screen for ADRs in EHR data. This includes developing reliable measures of AEs in EHR data, which is more or less challenging depending on the AE. Data categories that can be used to determine AEs are, for example, laboratory values defined by Logical Observation Identifiers Names and Codes (LOINC) or diagnoses defined by the International Statistical Classification of Diseases and Related Health Problems (10th Revision).^47^, ^48^ However, determining an appropriate combination of these data categories to represent the AE with high accuracy is challenging. For example, the incidence and prevalence of delirium will be underestimated if determined by ICD‐10‐coded diagnoses alone.^49^, ^50^ In addition, the real‐time availability of the selected data category in the EHR must be considered. In a parallel project, we are also working on these challenges of AE detection.

### Conclusions

The systematic categorization of the provided set of content‐validated drug‐event pairs as indicators of clinically important inpatient ADRs facilitates their future application in clinical practice (e.g., decision support), clinical surveillance (e.g., quality indicators) and research (e.g., outcome measures). Depending on whether sensitivity or specificity is more important, drug‐event pairs at least possible or only at least probable indicating an ADR can be used. As this consensus process is embedded in the POLAR_MI project, the developed indicators will be operationalized and implemented in EHR data of university hospitals throughout Germany to determine the prevalence of potential ADRs, support the efficient use of pharmacist resources, and develop risk models predicting ADRs.^23^

## FUNDING

The work on “Drug‐Event Pairs as Indicators for the Detection of Adverse Drug Reactions during Hospitalization in Routinely Collected Electronic Data Sources” within the overarching Use Case of the German Medical Informatics Initiative “POLAR_MI—POLypharmacy, Drug interActions, Risks” is supported by the German Federal Ministry of Education and Research (BMBF, 01ZZ1910H and 01ZZ1910L).

## CONFLICT OF INTEREST

The authors declared no competing interests for this work. The funders had no role in the design of the study; in the collection, analyses, or interpretation of data; in the writing of the manuscript; or in the decision to publish the results.

## AUTHOR CONTRIBUTIONS

A.M.W. wrote the manuscript; A.M.W., A.H., T.D., and U.J. designed the research; A.M.W., A.H., W.F., C.W., T.D., and U.J. performed the research; A.M.W. and U.J. analyzed the data.

## Supporting information


Data S1.

Data S2.

Data S3.

Data S4.

Data S5.

Data S6.
