# Supplementary material for: Drug‐Event Pairs as Indicators for the Detection of Adverse Drug Reactions during Hospitalization in Routinely Collected Electronic Data Sources
Source: Clin Pharmacol Ther. 2025 Mar 18;117(6):1811–9. doi: 10.1002/cpt.3635 (PMC12087692; doi:10.1002/cpt.3635)
Supplement: Supplementary file 1 — Data S1. [file CPT-117-1811-s003.pdf]

Journal: Clinical Pharmacology & Therapeutics

Drug-Event Pairs as Indicators for the Detection of Adverse Drug Reactions during Hospitalization in Routinely Collected Electronic Data Sources

SUPPLEMENT S1: Comprehensive literature search

Anna Maria Wermund[1], Annette Haerdtlein[2], Wolfgang Fehrmann[1], Clara Weglage[2], Tobias Dreischulte[2] and Ulrich Jaehde[1]*

[1] Department of Clinical Pharmacy, Institute of Pharmacy, University of Bonn, Bonn, Germany
[2] Institute of General Practice and Family Medicine, LMU University Hospital, LMU Munich, Munich, Germany
*Corresponding author. E-mail: u.jaehde@uni-bonn.de

Literature search procedure

The literature search was carried out by four different researchers (AMW, AH, CW, WF) from two different centers.

For each adverse drug reaction, we searched for systematic reviews published between 2000 and the search date in 2021. In addition, we looked for any type of review published in a peer-reviewed journal between 2010 and 2021. If there was insufficient evidence, we searched for all types of reviews published since 2000. The literature search was supplemented by the second edition of Anne Lee's book 'Adverse drug reactions' [30].

Inclusion and exclusion criteria

Table S1-1 Inclusion and exclusion criteria

	Inclusion criteria	Exclusion criteria
**E1:** Study design	Any kind of review in a peer reviewed journal	○ Simulation studies<br>○ Surveys<br>○ Experimental and observational studies
**E2:** Study population	Patients ≥ 18 years	○ Focus on patients with a specific disease/drug intake<br>○ Focus on neonates and children
**E3:** Setting	Any setting	○ Clinics for Traditional Chinese Medicine or similar health care services
**E4:** Evaluation*	**General description of potentially causative drugs for the specific event.**<br>A broad overview of potentially causative drugs for the specific event is provided.	○ Focus on a specific drug or class of drugs (e.g. review on paracetamol causing upper gastrointestinal bleeding)<br>○ Focus on herbal medicines or dietary supplements<br>○ Focus on medical devices and other aids (e.g. latex gloves)

* For the events "bleeding of the upper gastrointestinal tract (GIT)" and "bleeding outside the GIT", insufficient evidence was found in reviews that gave a broad overview of potentially causative drugs. For these events, reviews focusing on a specific drug or class of drugs were also included.

Search strategy and results

ADE/ADR drug related side effects and adverse reactions [MeSH Terms] OR drug-induced[All Fields] OR drug-related[All Fields] OR "adverse drug reaction"[All Fields] OR "adverse drug reactions"[All Fields] OR "adverse drug event"[All Fields] OR "adverse drug events"[All Fields] OR "adverse drug effect"[All Fields] OR "adverse drug effects"[All Fields]

AND

Specific term for each adverse event (see Table 2)

AND

Review [Filter] OR systematic review [Filter]

Language: English and German

Database: MEDLINE® (PubMed)

Table S1-2 Search strategies and results for each adverse drug reaction (Abbreviations: GIT = Gastrointestinal tract, R = Review; SR = Systematic review)

Adverse drug reaction	Search term	Time period	Number of results (number included)	Further literature considered
Rhabdomyolysis	rhabdomyolysis[MeSH] OR rhabdomyolysis [All Fields]	SR: 2000 – 05/2021<br>R: 2000 – 05/2021	SR: 10 (0)<br>R: 106 (7)	Search rhabdomyolysis AND review [Filter]: 629 (2)
Acute kidney injury*	Systematic review: acute kidney injury[MeSH] OR “acute kidney injury” OR “acute kidney injuries” OR “acute renal injury” OR “acute renal injuries” OR “acute renal insufficiency” OR” acute renal insufficiencies” OR “acute kidney insufficiency” OR “acute kidney insufficiencies” OR “acute kidney failure” OR “acute kidney failures” OR “acute renal failure” OR “acute renal failures” NOT COVID-19[MeSH]<br><br>Review: acute kidney injury [MeSH] NOT COVID-19[MeSH]	SR: 2010 – 06/2021<br>R: 2010 – 07/2021	SR: 804 (0)<br>R: 151 (16)	
Hypoglycemia	“hypoglycemia” OR "low blood sugar" OR “hypoglycaemia” OR “hypoglycemic” OR “hypoglycaemic” OR "hypoglycemic drugs" OR "hypoglycemic drug" OR "hypoglycemic agent" OR "hypoglycemic agents" OR “hypoglycemics” OR “hypoglycaemics” OR "hypoglycemic effect" OR "hypoglycemic effects" OR "hypoglycaemic drugs" OR "hypoglycaemic drug" OR "hypoglycaemic agent" OR "hypoglycaemic agents" OR "hypoglycaemic effect" OR "hypoglycaemic effects" OR hypoglycemia[MeSH] OR agents, hypoglycemic[MeSH]	SR: 2000 – 04/2021<br>R: 2007 – 04/2021	SR: 37 (2)<br>R: 284 (6)	
Anaphylaxis	"anaphylactic shock" OR "anaphylaxis" OR "anaphylactic reaction" OR "anaphylactic reactions" OR "anaphylactoid reaction" OR "anaphylactoid reactions" OR anaphylaxis[MeSH]	SR: 2000 – 05/2021<br>R: 2010 – 05/2021	SR: 34 (1)<br>R: 385 (37)	Hand search of selected publications: + 1 publication

Adverse drug reaction	Search term	Time period	Number of results (number included)	Further literature considered
Delirium	delirium OR deliriums OR delirium[MeSH]	SR: 2000 – 05/2021<br>R: 2000 – 05/2021	SR: 13 (2)<br>R: 108 (10)	Hand search of selected publications: + 2 publications
Hyperkalemia	hyperkalemia[MeSH] OR hypoaldosteronism[MeSH] OR hyperkalemia OR hyperpotassemia OR hyperkalaemias OR hyperkalaemia OR hyperpotassaemia OR hypoaldosteronisms OR hypoaldosteronism OR hyperkalemias OR hyperpotassemias	SR: 2000 – 04/2021<br>R: 2000 – 04/2021	SR: 2 (0)<br>R: 41 (5)	
Serotonin syndrome	serotonin syndrome[MeSH] OR „serotonin syndrome“ OR „serotonin toxicity“	SR: 2000 – 07/2021<br>R: 2010 – 07/2021	SR: 12 (1)<br>R: 99 (6)	
Bleeding outside the GIT	hemorrhage[MeSH] OR hematoma[MeSH] OR “hemorrhage” OR "haemorrhage" OR "bleeding" OR "hematoma" OR "haematoma" NOT gastrointestinal hemorrhage[MeSH] NOT "Gastrointestinal hemorrhage" NOT "Gastrointestinal bleeding"	SR: 2000 – 08/2021<br>R: 2010 – 08/2021	SR: 62 (12)<br>R: 352 (44)	
Agranulocytosis and neutropenia	neutropenia[MeSH] OR agranulocytosis[MeSH] OR acquired agranulocytosis [Supplementary Concept] OR neutropenia OR agranulocytosis OR granulocytopenia OR agranulosis	SR: 2000 – 07/2021<br>SR: 2010 – 07/2021	SR: 70 (1)<br>R: 172 (13)	Hand search of selected publications: + 3 publications
Stevens-Johnson Syndrome (SJS)/ Toxic Epidermal Necrolysis (TEN)	stevens-johnson syndrome[MeSH] OR stevens-johnson syndrome OR scalded skin syndrome OR toxic epidermal necrolysis OR lyell syndrome OR lyell’s syndrome	SR: 2000 – 04/2021<br>R: 2010 – 04/2021	SR: 41 (1)<br>R: 431 (14)	
Bleeding of the upper GIT **	upper gastrointestinal tract/drug effects [MeSH Major Topic] OR gastrointestinal hemorrhage[MeSH] OR "gastrointestinal hemorrhage” OR "gastrointestinal hemorrhages" OR "gastrointestinal bleeding"	SR: 2000 – 07/2021<br>R: 2010 – 07/2021	SR: 119 (33)<br>R: 446 (47)	

Adverse drug reaction	Search term	Time period	Number of results (number included)	Further literature considered
Hyponatremia	hyponatremia[MeSH] OR hyponatremia OR hyponatraemia OR hyponatraemias OR hyponatremias	SR: 2000 – 04/2021<br>R: 2000 – 04/2021	SR: 6 (0)<br>R: 58 (9)	

If no search field is specified, the default is [All Fields].

***** In order to keep the number of hits to a manageable level, the search strategy for ADRs was adapted**:** ("drug related side effects and adverse reactions"[MeSH Terms] OR "drug-induced"[Title/Abstract] OR "drug-related"[Title/Abstract] OR "adverse drug reaction"[Title/Abstract] OR "adverse drug reactions" [Title/Abstract] OR "adverse drug event"[Title/Abstract] OR "adverse drug events"[Title/Abstract] OR "adverse drug effect"[Title/Abstract] OR "adverse drug effects"[Title/Abstract])

** The following MeSH Terms were added to the ADE/ADR search term: Risk Factors, Risk Assessment

Table S1-3 Summary of included reviews

Adverse drug reaction	Number of publications	Book from Anne Lee	Systematic reviews	Reviews	Further literature
Rhabdomyolysis	9	yes	1	8	Drug-induced Rhabdomyolysis Atlas (DIRA) [38]
Acute kidney injury	16	yes	0	16	
Hypoglycemia	8	yes	2	6	
Anaphylaxis	39	no	1	38	
Delirium	14	yes	3	11	Anticholinergic burden (ACB) score by Kiesel et al. [39]
Hyperkalemia	5	no	0	5	
Serotonin syndrome	7	yes	1	6	
Bleeding outside the GIT	56	no	12	44	
Agranulocytosis and neutropenia	17	yes	1	16	
SJS/TEN	15	yes	1	14	
Bleeding of the upper GIT	80	yes	33	47	
Hyponatremia	9	no	0	9	

Abbreviations: GIT: Gastrointestinal tract; SJS/TEN: Stevens-Johnson Syndrome/Toxic Epidermal Necrolysis
