# Supplementary material for: Drug‐Event Pairs as Indicators for the Detection of Adverse Drug Reactions during Hospitalization in Routinely Collected Electronic Data Sources
Source: Clin Pharmacol Ther. 2025 Mar 18;117(6):1811–9. doi: 10.1002/cpt.3635 (PMC12087692; doi:10.1002/cpt.3635)
Supplement: Supplementary file 2 — Data S2. [file CPT-117-1811-s002.pdf]

Journal: Clinical Pharmacology & Therapeutics

Drug-Event Pairs as Indicators for the Detection of Adverse Drug Reactions during Hospitalization in Routinely Collected Electronic Data Sources

SUPPLEMENT S2: Example of the assessment form

Anna Maria Wermund[1], Annette Haerdtlein[2], Wolfgang Fehrmann[1], Clara Weglage[2], Tobias Dreischulte[2] and Ulrich Jaehde[1]*

[1] Department of Clinical Pharmacy, Institute of Pharmacy, University of Bonn, Bonn, Germany
[2] Institute of General Practice and Family Medicine, LMU University Hospital, LMU Munich, Munich, Germany
*Corresponding author. E-mail: u.jaehde@uni-bonn.de

Adverse event	Drug class	Drugs mentioned in the literature	Rating scale	Comments	Evidence
			**ADR Indicator**<br>Imagine an average patient during hospital stay developing hyperkalemia:<br>How likely is it that the listed medication significantly contributed to the adverse event, so that you would **assume** an **adverse drug reaction**?<br>0 = No comment<br>1 = Unlikely<br>2 = Possible<br>3 = Probable<br>4 = Certain	**Adjustment of drug classes (optional)**<br>In my opinion, there are drugs in this group that differ from the others in a meaningful way, so they should be assessed separately. These drugs are:	**Please note:** The precise risk of hyperkalemia with the listed drugs and drug classes is difficult to calculate because the risk for developing hyperkalemia increases with declining renal function. In addition, many of the listed drugs are commonly encountered in patients with various medical conditions that potentially contribute to hyperkalemia (e.g. renal failure, diabetes, heart failure, cirrhosis, hypoaldosteronism), making it difficult to calculate precise drug-induced incidences [5]. **The following applies to all drug classes: The risk is higher in the presence of a predisposing condition [1-5].**
Hyperkalemia	ACE Inhibitors	**Whole group**			**Mechanism of action**<br>*Reduced aldosterone secretion [1,3-5] / Impaired renal potassium excretion [4]*<br>Blockade of angiotensin II synthesis with decrease of aldosterone secretion. Impaired delivery of sodium to the distal nephron [1,4,5]. Reduced renal perfusion [3,4].<br><br>**Empirical evidence**<br>Reported percentage of hyperkalemia resulting from the medication: 8-37 % [1,3,4]. In a retrospective study investigating drug-associated life-threatening hyperkalemia, 47.1 % of cases were associated with an ACE inhibitor [1].<br>The incidence seems to be relatively low in patients with normal renal function/without predisposing factors. Serum potassium rarely increases by more than 0.5 mmol/L [1].<br>The risk appears to be proportional to the degree of renal insufficiency, but the serum potassium level can also increase significantly in patients with only moderate renal insufficiency [4]. In clinical trials, the risk of hyperkalemia with ACE inhibitor monotherapy in patients without predisposing factors is low (circa 2%-6%) and the absolute increases in serum levels are small [1,5].<br>However, the rates of hyperkalemia reported in clinical trials may not represent the risk in clinical practice, because most patients targeted for renovascular benefits of these drugs (such as those with diabetes, renal failure, or heart failure) are at high risk of developing hyperkalemia [1,3]. Hyperkalemia may occur in up to 10 % of outpatients within a year of commencing treatment with an ACE inhibitor [1].
Hyperkalemia	Angiotensin II Receptor Blockers (ARBs)	**Whole group**			**Mechanism of action**<br>*Reduced aldosterone secretion [1,3-5] / Impaired renal potassium excretion [4]*<br>Competitive binding to the angiotensin II receptor with decrease of aldosterone synthesis [1,5]; Reduced renal perfusion [3,4].<br><br>**Empirical evidence**<br>Reported percentage of patients who develop hyperkalemia: 2-7 % [4].<br>The incidence of hyperkalemia seems to not differ between ACE inhibitors and ARB (1.3 % with ACE inhibitors vs. 1.5 % with losartan) [1,3]. Although hyperkalemia associated with ARBs is rare in patients without risk factors, it is estimated to be 2–31 % in high-risk patients [1].
Hyperkalemia	Direct Renin Inhibitors	**Whole group**<br>Especially named in literature:<br>Aliskiren			**Mechanism of action**<br>*Reduced aldosterone secretion [1,5]*<br>Inhibition of the conversion of angiotensinogen to angiotensin I with decrease of aldosterone formation [1,5].<br><br>**Empirical evidence**<br>In a clinical trial, the proportion of patients with hyperkalemia was significantly higher in the aliskiren group than in the placebo group (11.2 vs. 7.2 %). In addition, hyperkalemia was the most common adverse event leading to the discontinuation of this study drug [1]. However, in a pooled analysis of seven randomized, double-blind studies including patients with hypertension treated with aliskiren, the incidence of hyperkalemia was similar to placebo [1]. The incidence of hyperkalemia is similar to that with ARB monotherapy (3.6 vs. 3.3 %, respectively) [1].
Hyperkalemia	Aldosteron Antagonists	**Whole group**<br>Especially named in literature:<br>Spironolactone, Eplerenone			**Mechanism of action**<br>*Tubular resistance to the action of aldosterone [1,3-5] / Impaired renal potassium excretion [4]*<br>Blockade of mineralocorticoid receptors [1,3-5].<br>In addition, the mechanism probably also involves extrarenal compensatory mechanisms: Decreased translocation of extracellular K+ to intracellular environments, decreased gastrointestinal secretion of potassium [1].<br><br>**Empirical evidence (Spironolactone, Eplerenone)**<br>Average raise of plasma potassium: 0.2–0.3 mmol/L (therapeutic dose) [1].<br>Spironolactone may also induce life-threatening hyperkalemia even in the presence of a normal glomerular filtration rate [1].<br>Similar to ACE inhibitors and ARBs, hyperkalemia occurrence is low in large controlled clinical trials, whereas in clinical practice a higher incidence was observed because of simultaneous use of potassium-altering medications and multiple diseases [1,3]. Aldosterone antagonists are frequently administered in combination to treat arterial hypertension or heart failure [1]. In the randomized Aldactone Evaluation Study, serious hyperkalaemia occurred in only 2 % of patients. By contrast, population based time-series analyses have demonstrated significant increases in rates of hospitalisation and mortality from hyperkalaemia after the use of aldosterone antagonists was recommended whenever possible to RAAS blockade and beta blockers [1, 3].<br><br>**Additional information**<br>Dose dependent [1,5].
Hyperkalemia	ENaC Blockers	**Whole group**<br>Especially named in literature:<br>Amiloride, Triamterene			**Mechanism of action**<br>*Tubular resistance to the action of aldosterone [1] / Impaired renal potassium excretion [4]*<br>Blockade of luminal sodium channels [1,3-5] with reduced mineralocorticoid activity due to resistance to the action of aldosterone action in the kidney [3].<br><br>**Empirical evidence**<br>Reported percentage of patients who develop hyperkalemia: 2-19 % [4]. Reported percentage of hyperkalemia resulting from the medication: 9-21 % [4].<br>Moderate to severe hyerkalemia has been reported in 4 % to 19 % of patients treated with ENaC Blockers [4].<br>Case reports about severe hyperkalemia can be found. [1].<br><br>**Additional information**<br>Dose dependent [5].
Hyperkalemia	Beta-blocking Agents	**Whole group**			**Mechanism of action**<br>*Inducing of transmembrane potassium movement [1,3,4]*<br>Decrease activity of Na^{+}/K^{+}-ATPase pump and renin release [1,4].<br><br>**Empirical evidence**<br>Reported percentage of patients who develop hyperkalemia: 1-5 % [4,5]. Reported percentage of hyperkalemia resulting from the medication (with other contributing factors): 4-17 % [1,4].<br>Moderate increases in serum potassium concentrations (circa 0.3 mmol/L), rarely severe [1,4]. In patients undergoing cardiopulmonary bypass procedure or with end-stage renal disease serum potassium values can increase by 1 mmol/L or more [1].<br>Hyperkalemia was mainly seen with nonselective rather than with cardio-selective beta-blockers [1,5].

Hyperkalemia	Calcium Channel Blockers	**Whole group**<br>Especially named in literature:<br>Verapamil, Diltiazem, Amlodipine, Nifedipine			**Mechanism of action**<br>Remains uncertain [1].<br>Inhibition of adrenal aldosterone biosynthesis; Reduction in aldosterone secretion [5].<br>*Verapamil:* May decrease potassium movement from the extracellular to the intracellular space by blocking calcium channels [1].<br><br>**Empirical evidence**<br>Very sporadic reports [1,5]. Chronic kidney disease, hypoaldosteronism and other concomitant hyperkalemia-inducing drugs are usually present.<br>Majority of cases associated with verapamil [1,5].
Hyperkalemia	NSAIDs	**Whole group** incl. COX2-Inhibitors			**Mechanism of action**<br>*Reduced aldosterone secretion [1,3,4] / Impaired renal potassium excretion [3,4]*<br>Decrease of prostaglandin-mediated renin release, renal blood flow and glomerular filtration rate [1,4,5] leading to hyporeninaemic hypoaldosteronism [3,4,5].<br>May cause direct renal toxicity [5].<br><br>**Empirical evidence**<br>Reported percentage of patients who develop hyperkalemia: 10-46 % [4]. Reported percentage of hyperkalemia resulting from the medication: 9-18 % [4].<br>NSAIDs have been reported to cause hyperkalemia in patients with or without renal insufficiency [1]. May be more common in cardiac patients [5].<br>In patients with normal kidney function, the mean increase in plasma potassium is typically around 0.2 mmol/L [1]. In patients with CKD, elevations in plasma potassium can exceed 1 mmol/L. Although the degree of hyperkalemia is often mild, it can be sufficiently severe to cause cardiac arrest and death [1]. The risk of hyperkalemia varies among individual NSAIDs, ranging from high risk for indomethacin to low risks for salicylates and the other NSAID groups [1,4]. Up to 46 % of hospital patients treated with indomethacin develop an increase in serum potassium levels or hyperkalemia [4]. A retrospective cohort study performed by Aljadhey et al. suggested that selective cyclooxygenase (COX)-2 inhibitors may pose a greater risk of hyperkalemia than nonselective NSAIDs (increase in serum potassium of 0.15 mmol/l versus nonselective NSAIDs). [1]<br><br>**Additional information**<br>The decrease in potassium secretion begins to occur with the first dose of NSAID [1].
Hyperkalemia	Heparin and Derivatives	**Whole group**<br>Especially named in literature:<br>Unfractionated heparin (UFH), Low molecular weight heparin (LMWH)			**Mechanism of action**<br>*Reduced aldosterone secretion [1,3,5] / Impaired renal potassium excretion [4]*<br>Inhibition of aldosterone production by reduction in both the number and affinity of the angiotensin II receptors [1,2,4,5].<br>Inhibition of the final enzymatic steps of aldosterone formation [4].<br>Excess anticoagulation may also, in rare circumstances, precipitate adrenal hemorrhage and induce adrenal insufficiency [1].<br><br>**Empirical evidence**<br>Reported percentage of patients who develop hyperkalemia: 8-17 % [4]. Reported percentage of hyperkalemia resulting from the medication: 1-20 % [4].<br>Heparin-induced hyperkalemia (HIH) might occur in approximately 7–8 % of heparin-treated patients [1]. Elevations in serum potassium have ranged from 0.2 to 1.7 mmol/L [1,4]. It may be seen after either i.v. or s.c. administration. Most patients remain asymptomatic [1]. However, patients with preexisting defects in potassium homeostatis or patients receiving prolonged heparin therapy are especially predisposed to HIH [1,4].<br>Hyperkalemia is not common but there are many case reports [5].<br><br>**Additional information**<br>Dose dependent, occurs rapidly following initiation of therapy [1].
Hyperkalemia	Calcineurin Inhibitors	**Whole group**<br>Especially named in literature:<br>Tacrolimus, Ciclosporin			**Mechanism of action**<br>*Reduced aldosterone secretion [1,3-5] / Impaired renal potassium excretion [4]*<br>Decrease aldosterone synthesis and Na^{+}/K^{+}-ATPase pump activity [1,4,5].<br>An aldosterone resistance secondary to decreased transcription of human mineralocorticoid receptors on peripheral blood leukocytes is also reported [1].<br>*Ciclosporin* may also induce a chloride channel shunt that impairs the electrochemical driving force for potassium secretion [4]. It also inhibits apical secretory potassium channel activity in principal cells. Further it can cause acute, transient hyperkalemia by increasing potassium efflux from cells [4].<br><br>**Empirical evidence**<br>Reported percentage of patients who develop hyperkalemia: 11-44 % (C.), 15-53 % (T.) [4]. Reported percentage of hyperkalemia resulting from the medication: 5-28 % (C.), 8-28 % (T.) [4].<br>Mild and uncomplicated hyperkalemia is commonly observed in patients treated with calcineurin inhibitors, especially transplant recipients (mainly renal transplant patients) [1,4]. 44-73 % of transplant recipients develop hyperkalemia [1]. The increased incidence may also be due the development of impaired kidney function in individuals chronically treated with these medications. However, hyperkalemia may occur despite adequate kidney function [1].
Hyperkalemia	**Certain** Antiinfectives	**Certain Antiinfectives:**<br>Pentamidine, Cotrimoxazole (Trimethoprim/Sulfamethoxazole)			**Mechanism of action**<br>Pentamidine: Blockade of luminal sodium channels [1,3-5].<br>Trimethoprim: Blockade of luminal sodium channels [1,3-5] and inhibition of Na+/K+-ATPase [1].<br><br>**Empirical evidence**<br>*Pentamidine:* Pentamidine administration appears to be causally related to life-threatening hyperkalemia in the presence of a mild to severe renal insufficiency [1,2,4]. Reported percentage of patients who develop hyperkalemia: 5-24 % [4]; Reported percentage of hyperkalemia resulting from the medication 0-5 % [4].<br>*Trimethoprim:* An increase in serum potassium level, which ranges from 0.36 to 1.21 mmol/L or greater, occurs in most patients who receive trimethoprim [1]. Reported percentage of patients who develop hyperkalemia: 6-21 % [4]; Reported percentage of hyperkalemia resulting from the mediaction 14-29 % [4].
Hyperkalemia	Potassium-Containing Agents	**Whole group**<br>For example: Penicillin G, Potassium Citrate, Potassium supplements (oral or i.v.), Salt substitutes			**Mechanism of action**<br>*Potassium Input*<br><br>**Empirical evidence**<br>Percentage of patients who develop hyperkalemia 3-24 % [4]. Reported percentage of hyperkalemia resulting from the medication 11-58 % [4].<br>Potassium administration alone rarely induces hyperkalemia in the absence of an underlying defect in potassium homeostasis [1,2,4]. Incidence is quite high in patients with renal dysfunction [5].<br>The Boston Collaborative Drug Surveillance Program demonstrated a 3.6 % incidence of hyperkalemia among 4,921 patients taking physician-prescribed potassium supplements [1,4]. Other studies revealed that potassium supplements cause or contribute to hyperkalemia in approximately 15 % to 40 % of hospitalized patients [4].<br>Prolonged or excessive ingestion of potassium citrate may lead to severe hyperkalemia [1].<br>Penicillin G is usually administered as potassium salt (contains 1.7 mmol of potassium per million units). It can significantly alter potassium balance when given in very high doses. Severe hyperkalemia with cardiac arrest caused by penicillin is reported. Rapid intravenous infusion or oral administration of large amounts of semisynthetic penicillin derivatives may be potentially dangerous in patients with diabetes and renal insufficiency [1].
Hyperkalemia	Miscellaneous Drugs	Digoxin<br>Mannitol<br>Suxamethonium<br>Propofol<br><br>Ketoconazole<br>Drospirenone<br><br>Magnesiumsulfates			**Mechanism of action**<br>***Digoxin, Mannitol, Suxamethonium, Propofol*** : Drugs inducing transmembrane potassium movement [1,3-5].<br>***Drospirenone, Ketoconazole:*** Tubular resistance to the action of aldosterone [1-5].<br>***Magnesiumsulfates:*** Unknown mechanism.<br><br>**Empirical evidence**<br>***Digoxin:*** Resulting from both acute and chronic toxicity. Therapeutic digoxin levels do not lead to hyperkalemia unless there are other predisposing factors [1,4,5]. Overdose may cause hyperkalemia that can be fatal [4]. Rarely, hyperkalemia develops in patients who have therapeutic or mildly increased digoxin levels if other risk factors for impaired potassium handling are present [4].<br><br>***Suxamethonium:*** It causes an increase in serum potassium up to 1 mmol/L [1]. In normal subjects, the mean plasma potassium level increased by 0.5 mEq/liter within 3 to 5 minutes after i.v. application [1,4]. Marked hyperkalemia has been reported in patients with burns, occult myopathies, muscle injury, trauma, neuromuscular disease and severe infection [1,4,5].<br><br>***Ketoconazole, Drospirenone, Propofol, Magnesiumsulfates, Mannitol:*** Very sporadic reports [1,5].

Hyperkalemia	Combination of two drugs you rated as possible (=2)				**Empirical evidence**<br>Drug interactions are a common problem in the setting of hyperkalemia.<br>**For all drug classes, it has been stated in the literature that the risk is greater when they are used concomitantly with other potassium-altering medications [1-5].**<br>Many of the listed drugs are commonly combined, and those combinations may potentiate the risk of hyperkalemia [1,5]. An important example includes combining an ACE inhibitor or an ARB with a NSAID. Both of these drug classes have been shown to potentiate the risk of hyperkalemia separately. In addition, both drug classes potentiate the risk of developing renal dysfunction [5].<br>Several listed drugs are combined for the treatment of congestive heart failure or hypertension [1,3].
Hyperkalemia	Combination of two drugs you rated as probable (=3)				**Empirical evidence**<br>Drug interactions are a common problem in the setting of hyperkalemia.<br>**For all drug classes, it has been stated in the literature that the risk is greater when they are used concomitantly with other potassium-altering medicatons [1-5].**<br>Many of the listed drugs are commonly combined, and those combinations may potentiate the risk of hyperkalemia [1,5]. An important example includes combining an ACE inhibitor or an ARB with a NSAID. Both of these drug classes have been shown to potentiate the risk of hyperkalemia separately. In addition, both drug classes potentiate the risk of developing renal dysfunction [5].<br>Several listed drugs are combined for the treatment of congestive heart failure or hypertension [1,3].

Further questions

Inclusion of medicines not previously considered

Consider the drugs listed in the next column: If you want to add one or more drugs as an indicator, please tick them.	Azole Antifungals	☐
	Amphotericin B	☐
	Dabigatran	☐
	Ethinyl estradiole	☐
	Hydroxycarbamide	☐
	Insulin	☐
	Nafarelin	☐
	Octreotide	☐
	Omeprazole	☐
	Thalidomid	☐
	Zoledronic acid	☐
	**Other**	☐
		Please define here which drug you want to add

References
[1] Ben Salem C et al. Drug-induced hyperkalemia. Drug Saf. 2014; 37 (9):677-92
[2] Kokot F et al. Drug-induced abnormalities of potassium metabolism. Pol Arch Med Wewn. 2008; 118 (7-8):431-4
[3] Nyirenda et al. Hyperkalaemia. BMJ. 2009; 339:b4114
[4] Perazella MA. Drug-induced hyperkalemia: old culprits and new offenders. Am J Med. 2000; 109 (4):307-14
[5] Wooten JM et al. A Brief Review of the Pharmacology of Hyperkalemia: Causes and Treatment. South Med J. 2019; 112 (4):228-233
