# Supplementary material for: Drug‐Event Pairs as Indicators for the Detection of Adverse Drug Reactions during Hospitalization in Routinely Collected Electronic Data Sources
Source: Clin Pharmacol Ther. 2025 Mar 18;117(6):1811–9. doi: 10.1002/cpt.3635 (PMC12087692; doi:10.1002/cpt.3635)
Supplement: Supplementary file 3 — Data S3. [file CPT-117-1811-s006.pdf]

Journal: Clinical Pharmacology & Therapeutics

Drug-Event Pairs as Indicators for the Detection of Adverse Drug Reactions during Hospitalization in Routinely Collected Electronic Data Sources

SUPPLEMENT S3: Pre-test interview guide

Anna Maria Wermund[1], Annette Haerdtlein[2], Wolfgang Fehrmann[1], Clara Weglage[2], Tobias Dreischulte[2] and Ulrich Jaehde[1]*

[1] Department of Clinical Pharmacy, Institute of Pharmacy, University of Bonn, Bonn, Germany
[2] Institute of General Practice and Family Medicine, LMU University Hospital, LMU Munich, Munich, Germany
*Corresponding author. E-mail: u.jaehde@uni-bonn.de

Interview guide

Translated interview guide. Interviews were held in German.

Introduction and getting started

Thank you for taking the time to test our RAM process.

Using the RAM methodology, we aim to combine evidence and expert opinion to identify drug-event pairs as indicators to capture potential adverse drug reactions (ADRs) in routine hospital data. Building on the first stage of our RAM process [the previous consensus process], which identified clinically important adverse events with a high drug-relatedness, we will now identify indicators for ADRs consisting of a drug or drug class and the adverse event (drug-event pairs). For this purpose, the assessment form consists of potentially causative drugs for each ADR, collected through a comprehensive literature search.

Question: How long did it take you to complete the assessment form?

Comprehension and suitability of the assessment criteria for identifying indicators for the detection of ADRs

Step 1: Participants were presented with three different options

Validity (How do you rate the validity of the combination (adverse event + drug) as an indicator for an adverse drug reaction? 1 = Not valid; 2; 3; 4 = Valid)

1. How did you deal with this assessment criterion in general? Did you understand what is meant by a valid indicator?
2. Did you have problems with levels 2 and 3? Have you used them? Should the intermediate levels be specifically labelled or can you get by with just the „endpoint label“?
3. What **considerations** did you make when assessing "validity"? What **challenges** did you face during the assessment?

Causality (How do you estimate the strength of the causal link between the adverse event and the listed drug class? 1 = Very weak; 2 = Weak; 3 = Strong; 4 = Very strong)

1. How did you deal with this assessment criterion in general? Did you understand it?
2. Did you have problems with levels 2 and 3? Have you used them?
3. What **considerations** did you make when assessing "causality"? What **challenges** did you face during the assessment?

Clinical context (How important is it to discontinue the medication or to reduce the dose as a strategy to prevent further or repeated harm from this adverse event? 1 = Not important; 2 = Somewhat important; 3 = Important; 4 = Very important)

1. How did you generally deal with this assessment criterion embedded in a clinical context? Did you understand it?
2. Did you have problems with levels 2 and 3? Have you used them?
3. What **considerations** did you make when assessing the causal relationship within the clinical context? What **challenges** did you face during the assessment?

To conclude: Which of the three assessment criteria do you think is most appropriate for assessing the drug-event pairs as indicators of ADRs?

Step 2: Participants were presented with a modified criterion

ADR indicator

1. How did you deal with this assessment criterion in general? Did you understand what was to be assessed?
2. Did you use the intermediate levels 2 and 3? Did you have problems with them?
3. What **considerations** did you make during the assessment? What **challenges** did you face during the assessment?

Comprehension and suitability of drug classes

1. How did you deal with the drug classes in general? Were you able to cope with the grouping of the drug classes?
2. Which drug classes were unclear or difficult for you to assess? Please tell me the relevant drug classes and the ambiguities/difficulties you experienced.
   Could a more detailed categorisation contribute to greater clarity?
3. Did you base your assessment on a specific drug in the class or did you use all drugs in a class for your assessment?

Comprehension and suitability of additional questions

1. In general, how did you deal with the two questions regarding the inclusion of other drugs? Did you understand them?

Assessment of the evidence report

1. How helpful was the (attached) evidence report?
2. What other information would you like to have?
3. How did you incorporate the evidence into your evaluation?
4. What did you think of the position of the evidence in the assessment form? Would you have preferred a separate document?

Final question

Do you have any further suggestions regarding our RAM process?
