# Supplementary material for: Drug‐Event Pairs as Indicators for the Detection of Adverse Drug Reactions during Hospitalization in Routinely Collected Electronic Data Sources
Source: Clin Pharmacol Ther. 2025 Mar 18;117(6):1811–9. doi: 10.1002/cpt.3635 (PMC12087692; doi:10.1002/cpt.3635)
Supplement: Supplementary file 4 — Data S4. [file CPT-117-1811-s001.pdf]

Journal: Clinical Pharmacology & Therapeutics

Drug-Event Pairs as Indicators for the Detection of Adverse Drug Reactions during Hospitalization in Routinely Collected Electronic Data Sources

SUPPLEMENT S4: Excluded drugs and drug classes

Anna Maria Wermund[1], Annette Haerdtlein[2], Wolfgang Fehrmann[1], Clara Weglage[2], Tobias Dreischulte[2] and Ulrich Jaehde[1]*

[1] Department of Clinical Pharmacy, Institute of Pharmacy, University of Bonn, Bonn, Germany
[2] Institute of General Practice and Family Medicine, LMU University Hospital, LMU Munich, Munich, Germany
*Corresponding author. E-mail: u.jaehde@uni-bonn.de

Table S4 Excluded drugs and drug classes, listed below the drug classes to be assessed

**Adverse drug reaction**	**Excluded drugs and drug classes**
Hyperkalemia	• Azole antifungals<br>• Amphotericin B<br>• Dabigatran<br>• Ethinylestradiol<br>• Hydroxycarbamide<br>• Insulin<br>• Nafarelin<br>• Octreotide<br>• Omeprazole<br>• Thalidomide<br>• Zoledronic acid
Hyponatremia	• Antiarrhythmics<br>• Antibiotics<br>• Benzodiazepines<br>• Barbiturates<br>• Calcium channel blockers<br>• Everolimus<br>• Temsirolimus<br>• Insulin<br>• Pentamidine<br>• Miconazole<br>• Metformin<br>• Metoclopramide
Hypoglycemia	• Tyrosine kinase inhibitors<br>• Narcotics and other sedatives (Fentanyl)<br>• Monoamine oxidase inhibitors<br>• Terbutaline<br>• Adrenaline<br>• Atosiban<br>• Brimonidine tartrate<br>• Bupivacaine<br>• Darbepoetin alfa<br>• Heparin<br>• Latanoprost<br>• Methimazole (Thiamazole)

	• Naltrexone<br>• Pantoprazole<br>• Prednisolone<br>• Varenicline<br>• Infliximab<br>• Penicillamine<br>• Doxorubicin<br>• Busulfan<br>• Gemcitabine<br>• L-Asparaginase<br>• Methotrexate<br>• Paclitaxel<br>• Pemetrexed<br>• Irinotecan<br>• Rituximab<br>• Topotecan<br>• Carboplatin<br>• Olanzapine<br>• Zuclopenthixol<br>• Buprenorphine<br>• Tiopronin
Bleeding of the upper gastrointestinal tract	• Mirtazapine<br>• Bupropion
Bleeding outside the gastrointestinal tract	• Amphotericin B<br>• Propranolol<br>• Paracetamol<br>• Interferon-alfa<br>• Phenothiazine<br>• Thioxanthene<br>• Amiodarone<br>• Propafenone<br>• Penicillamine<br>• Ranitidine<br>• Fibrinolytics<br>• Corticosteroids (intranasal)
Stevens-Johnson syndrome (SJS) and toxic epidermal necrolysis (TEN)	• Fexofenadine<br>• Antimalarials (Quinine)<br>• Pyrazolones (Metamizole)<br>• Antiemetics (Domperidone, Ondansetron)<br>• Protease inhibitors<br>• Thiazide diuretics<br>• Ranitidine<br>• Sulfonylurea antidiabetics<br>• Insulin<br>• Phenothiazines<br>• Calcium channel blockers<br>• Angiotensin-converting enzyme inhibitors<br>• Beta blockers
Anaphylaxis	• Angiotensin-converting enzyme inhibitors<br>• Anticonvulsants<br>• Fibrin sealants<br>• Epinephrine

	• Ranitidine<br>• Amphotericin B<br>• Cyclosporine<br>• Iron supplements<br>• Hydroxychloroquine
Serotonin syndrome	• Ergot alkaloids (Ergotamine, Methylergometrine)<br>• Clonazepam<br>• Alprazolam<br>• Levodopa<br>• Atomoxetine<br>• Hydroxyzine<br>• Propofol<br>• Haloperidol<br>• Promethazine
Agranulocytosis and neutropenia	• Pirenzepine<br>• Octreotide<br>• Torsemide
Acute kidney injury	• Anaesthetics<br>• Opioids (Methadone)<br>• Sodium glucose linked transporter 2 inhibitors<br>• Conjugated estrogens<br>• Tumour necrosis factor blockers<br>• Anticoagulants<br>• Uricosuric drugs<br>• Iron chelating agents<br>• Sodium phosphate purgatives<br>• Methyldopa<br>• Colchicine<br>• Heparin
Rhabdomyolysis	• Narcotics/Anaesthetics (Ketamine)<br>• Laxatives<br>• Serotonin antagonists<br>• Monoamine oxidase inhibitors<br>• Antimalarials<br>• Glycopyrrolate<br>• Trospium<br>• Pantoprazole<br>• Rabeprazole<br>• Interferon beta-1a<br>• Avelumab<br>• Urokinase<br>• Tenecteplase<br>• Methylphenidate<br>• Atomoxetine<br>• Mifepristone<br>• Metoclopramide<br>• Tolvaptan<br>• Chloral hydrate<br>• Tetrabenazine<br>• Cobicistat<br>• Albendazole<br>• Levodopa

	• Pramipexole<br>• Aliskiren<br>• Trientin-HCl<br>• Eprosartan<br>• Valsartan<br>• Telmisartan<br>• Irbesartan<br>• Candesartan
Delirium	• Alpha-blocking agents<br>• Calcium channel clockers (dihydropyridine type)<br>• Angiotensin-converting enzyme inhibitors<br>• Sympathomimetic agents
