# Supplementary material for: Drug‐Event Pairs as Indicators for the Detection of Adverse Drug Reactions during Hospitalization in Routinely Collected Electronic Data Sources
Source: Clin Pharmacol Ther. 2025 Mar 18;117(6):1811–9. doi: 10.1002/cpt.3635 (PMC12087692; doi:10.1002/cpt.3635)
Supplement: Supplementary file 5 — Data S5. [file CPT-117-1811-s004.pdf]

Journal: Clinical Pharmacology & Therapeutics

Drug-Event Pairs as Indicators for the Detection of Adverse Drug Reactions during Hospitalization in Routinely Collected Electronic Data Sources

SUPPLEMENT S5: Rating results

Anna Maria Wermund[1], Annette Haerdtlein[2], Wolfgang Fehrmann[1], Clara Weglage[2], Tobias Dreischulte[2] and Ulrich Jaehde[1]*

[1] Department of Clinical Pharmacy, Institute of Pharmacy, University of Bonn, Bonn, Germany
[2] Institute of General Practice and Family Medicine, LMU University Hospital, LMU Munich, Munich, Germany
*Corresponding author. E-mail: u.jaehde@uni-bonn.de

Table S5-1 Expert ratings of the first round

		Number of participants with rating							
Drug-event pairs sorted by event	Certain drugs	1	2	3	4	0	Median	Disagreement	Category
Hyperkalemia									
ACE inhibitors		0	3	6	1	0	3	yes	possible
ARBs		0	5	5	0	0	2.5	yes	possible
Direct renin inhibitors		0	4	5	0	1	3	yes	possible
Aldosterone antagonists		0	2	7	1	0	3	no	probable
ENaC blockers		1	4	5	0	0	2.5	yes	possible
Beta-blocking agents		7	1	1	0	1	1	no	unlikely
CCBs		10	0	0	0	0	1	no	unlikely
NSAIDs		2	7	1	0	0	2	no	possible
Heparin and derivatives		6	4	0	0	0	1	no	unlikely

Calcineurin inhibitors		2	6	2	0	0	2	no	possible
Certain anti-infectives	Pentamidine, Sulfamethoxazole/Trimethoprim (Cotrimoxazole)	2	6	1	0	1	2	no	possible
Potassium-containing agents		1	3	5	1	0	3	yes	possible
Miscellaneous drugs	Digoxin, Mannitol, Propofol, Ketoconazole, Drospirenone, Magnesium sulfate, Suxamethonium	5	3	0	1	1	1	no	unlikely
Two drugs rated as possible (=2)		0	2	7	0	1	3	no	probable
Two drugs rated as probable (=3)		0	0	8	1	1	3	no	probable
Hyponatremia									
Oncologicals		1	5	3	0	1	2	yes	possible
MAO inhibitors		4	4	1	0	1	2	no	possible
SSNRI / SSRI		0	3	7	0	0	3	yes	possible
Tricyclic and tetracyclic antidepressants		0	7	3	0	0	2	yes	possible
Antipsychotics		1	7	2	0	0	2	no	possible
Thiazides		0	1	8	1	0	3	no	probable
Other diuretics		0	2	8	0	0	3	no	probable
ACE inhibitors		5	4	1	0	0	1.5	no	unlikely
Anticonvulsants		1	6	3	0	0	2	yes	possible
Vasopressin and analogues		0	4	3	1	2	2.5	yes	possible
Certain laxatives	Macrogol, Sodium picosulfate	3	7	0	0	0	2	no	possible
NSAIDs		9	0	1	0	0	1	no	unlikely
Opiates		8	1	1	0	0	1	no	unlikely

Proton pump inhibitors		8	2	0	0	0	1	no	unlikely
Certain antibiotics	Ciprofloxacin, Linezolid, Rifabutin, Sulfamethoxazole/Trimethoprim	9	1	0	0	0	1	no	unlikely
Miscellaneous drugs	Amiodarone, Amlodipine, Bromocriptine, Bupropion, Immunoglobulin, Paracetamol, Propafenone, Theophylline, Tolbutamide, Voriconazole	8	2	0	0	0	1	no	unlikely
Two drugs rated as possible (=2)		0	5	5	0	0	2.5	yes	possible
Two drugs rated as probable (=3)		0	1	7	2	0	3	no	probable
Hypoglycemia									
Incretin therapy		4	4	2	0	0	2	no	possible
Classic insulin secretagogues (Sulfonylureas and Glinides)		0	0	6	3	1	3	no	probable
Non-insulinotropic antidiabetics		6	3	1	0	0	1	no	unlikely
Insulin		0	1	3	6	0	4	no	certain
ACE inhibitors		8	1	1	0	0	1	no	unlikely
Beta-blocking agents		5	5	0	0	0	1.5	no	unlikely
Certain ARBs	Losartan, Telmisartan, Valsartan	8	2	0	0	0	1	no	unlikely
Certain α2-receptor agonists	Clonidine, Dexmedetomidine	9	1	0	0	0	1	no	unlikely
Certain CCBs (Dihydropyridine type)	Nifedipine, Felodipine	10	0	0	0	0	1	no	unlikely
Certain serotonergic antidepressants	Venlafaxine, Sertraline, Fluoxetine, Imipramine, Maprotiline, Nortriptyline, Doxepin	10	0	0	0	0	1	no	unlikely
Certain anticonvulsants	Gabapentin, Phenytoin, Topiramate, Valproate	8	2	0	0	0	1	no	unlikely
Anti-malaria drugs		3	5	0	0	2	2	no	possible
Quinolones		5	5	0	0	0	1.5	no	unlikely

Certain other antibiotics	Ceftriaxone, Clarithromycin, Doxycycline, Tetracycline, Oxytetracycline, Isoniazid, Para-aminosalicylic acid, Pentamidine, Piperacillin-tazobactam, Sulfadiazine, Sulfamethoxazole/ Trimethoprim	8	2	0	0	0	1	no	unlikely
Azole antimycotics		9	1	0	0	0	1	no	unlikely
Certain NSAIDs	Ibuprofen, Indomethacin, Phenylbutazone, Salicylates, Piroxicam	6	4	0	0	0	1	no	unlikely
Certain antivirals	Amprenavir, Entecavir, Ganciclovir, Saquinavir, Stavudine, Zidovudine	7	3	0	0	0	1	no	unlikely
Certain growth hormones	IGF-I (Mecasermin), Somatotropin	3	5	1	0	1	2	no	possible
Miscellaneous drugs	Amiodarone, Atorvastatin, Bortezomib, Chlormadinone, Clonazepam Propoxyphene/Dextropropoxyphene, Donepezil, Imatinib, Haloperidol, Etanercept, Ethacrynic acid, Etomidate, Hydralazine, Lidocaine, IL-2, Interferons, Lenalidomide, Lithium, Levothyroxine, 6-Mercaptopurine, Mifepristone, Octreotide, Paracetamol, Ranitidine, Salbutamol, Selegiline, Tramadol	9	0	0	0	1	1	no	unlikely
Two drugs rated as possible (=2)		0	5	5	0	0	2.5	yes	possible
Two drugs rated as probable (=3)		0	0	7	2	1	3	no	probable
Bleeding of the upper GIT									
NSAIDs (non-selective)		0	3	6	1	0	3	yes	possible
Selective COX-2 inhibitors		1	6	3	0	0	2	yes	possible
Paracetamol		7	2	0	0	1	1	no	unlikely
Direct oral anticoagulants		0	2	6	2	0	3	no	probable
Vitamin K antagonists		0	3	5	2	0	3	yes	possible
Antiplatelet drugs		0	2	6	2	0	3	no	probable

CCBs		10	0	0	0	0	1	no	unlikely
SSRI		2	6	2	0	0	2	no	possible
Glucocorticoids		2	6	2	0	0	2	no	possible
Certain angiogenesis inhibitors	Bevacizumab, Erlotinib, Axitinib, Aflibercept	2	2	5	0	1	3	yes	possible
Two drugs rated as possible (=2)		0	5	4	1	0	2.5	yes	possible
Two drugs rated as probable (=3)		0	0	8	2	0	3	no	probable
Bleeding outside the GIT									
NSAIDs (non-selective)		2	3	4	0	1	2	yes	possible
Heparins		1	2	5	2	0	3	yes	possible
Vitamin K antagonists		0	1	7	2	0	3	no	probable
Direct oral anticoagulants		0	1	7	2	0	3	no	probable
Certain other anticoagulants	Fondaparinux, Argatroban, Bivalirudin	0	2	6	2	0	3	no	probable
Antiplatelet drugs (excl. Dipyridamole, Cilostazol)		1	2	6	1	0	3	yes	possible
Certain phosphodiesterase inhibitors	Dipyridamole, Cilostazol	2	4	2	0	2	2	no	possible
SSRI / SSNRI		4	6	0	0	0	2	no	possible
Certain angiogenesis inhibitors	Ranibizumab, Bevacizumab, Sorafenib	1	5	3	0	1	2	yes	possible
Certain anticancer drugs	Fludarabine, Cytarabine, Cyclophosphamide, Ifosfamide, Oxaliplatin, Ramucirumab, Ibrutinib, Acalabrutinib, Zanubrutinib, Gefitinib, Azathioprine/Mercaptopurine	1	6	3	0	0	2	yes	possible
Certain beta-lactams	*Penicillin's:* Penicillin, Ampicillin, Piperacillin *Cephalosporins:* Ceftriaxone	6	2	0	0	2	1	no	unlikely
Certain other antibiotics	Trimethoprim/Sulfamethoxazole, Vancomycin, Rifampicin, Mitomycin, Nitrofurantoine	7	2	0	0	1	1	no	unlikely

Certain antiepileptics	Phenytoin, Carbamazepine, Valproic acid	7	2	0	0	1	1	no	unlikely
Miscellaneous drugs	Amlodipine, Mirtazapine, Quinine	9	0	0	0	1	1	no	unlikely
Two drugs rated as possible (=2)		0	3	6	0	1	3	yes	possible
Two drugs rated as probable (=3)		0	1	6	3	0	3	no	probable
Stevens-Johnson syndrome and toxic epidermal necrolysis									
Certain antiepileptics	Carbamazepine, Phenytoin, Phenobarbital, Valproate, Lamotrigine, Oxcarbazepine, Levetiracetam	0	4	4	2	0	3	yes	possible
Antibiotics (excl. Sulphonamides and Antituberculotics)		0	7	3	0	0	2	yes	possible
Sulphonamides		0	3	7	0	0	3	yes	possible
Antituberculotics		2	7	1	0	0	2	no	possible
Nevirapine		0	3	5	0	2	3	yes	possible
Antiretroviral therapy		2	5	2	0	1	2	no	possible
Certain antimycotics	Fluconazole, Nystatin, Terbinafine	5	3	2	0	0	1.5	no	unlikely
NSAIDs + Paracetamol		2	7	1	0	0	2	no	possible
Certain diuretics	Furosemide, Acetazolamide	4	6	0	0	0	2	no	possible
Glucocorticoids		7	3	0	0	0	1	no	unlikely
Allopurinol		0	4	5	0	1	3	yes	possible
Certain antidepressants	Sertraline, Mirtazapine, Duloxetine	3	6	1	0	0	2	no	possible
Certain anticancer drugs	*Taxanes:* Docetaxel, Paclitaxel<br>*TKIs:* Afatinib, Imatinib, Sunitinib, Vandetanib,<br>*Checkpoint inhibitors:* Ipilimumab, Nivolumab, Pembrolizumab	4	6	0	0	0	2	no	possible

	Other: Cetuximab, Leflunomide, Lenalidomide, Tamoxifen, Thalidomide, Vemurafenib								
Miscellaneous drugs	Amlodipine, Chloroquine, Famotidine, Isotretinoin, Oseltamivir, Pantoprazole, Ramipril, Tramadol	5	3	1	0	1	1	no	unlikely
Two drugs rated as possible (=2)		1	7	1	1	0	2	no	possible
Two drugs rated as probable (=3)		1	1	4	3	1	3	no	probable
Anaphylaxis									
Beta-lactams		0	2	5	3	0	3	no	probable
Fluoroquinolones		2	5	2	1	0	2	yes	possible
Sulphonamides		0	6	2	2	0	2	yes	possible
Macrolides		3	5	1	1	0	2	no	possible
Tetracyclines		1	7	0	1	1	2	no	possible
Glycopeptide antibiotics		1	4	3	2	0	2.5	yes	possible
Certain other antibiotics	Clindamycin, Metronidazole, Rifampicin, Bacitracin, Gentamicin, Tobramycin	3	6	1	0	0	2	no	possible
Anaesthetic agents		3	6	1	0	0	2	no	possible
Local anaesthetics		1	5	4	0	0	2	yes	possible
Glucocorticoids		7	3	0	0	0	1	no	unlikely
Certain chemotherapy agents	*Taxanes:* Docetaxel, Paclitaxel<br>*Platinum compounds:* Carboplatin, Cisplatin, Oxaliplatin<br>*Topoisomerase II inhibitors:* Podophyllotoxins (Etoposide, Teniposid), Doxorubicin<br>*Other:* L-Asparaginase, Procarbazine, Cyclophosphamide, Cytarabine, Methotrexate, Leflunomide	1	7	2	0	0	2	no	possible

Contrast media		0	4	5	1	0	3	yes	possible
Neuromuscular blocking agents		0	3	5	1	1	3	yes	possible
NSAIDs		2	5	3	0	0	2	yes	possible
Opioids		1	9	0	0	0	2	no	possible
Certain other analgesics	Paracetamol, Metamizole (i.v.)	1	7	1	1	0	2	no	possible
Proton pump inhibitors		7	2	1	0	0	1	no	unlikely
Certain 5-HT3 antagonists	Ondansetron, Palonosetron	6	4	0	0	0	1	no	unlikely
Certain uterotonic drugs	Oxytocin, Dinoprostone	4	5	0	0	1	2	no	possible
Biologicals		1	4	3	2	0	2.5	yes	possible
Miscellaneous drugs	Abacavir, Atropine, Cetirizine, Heparin, Hyaluronidase, Mannitol, Neostigmine, Sugammadex, Tranexamic acid, Protamine	4	5	0	0	1	2	no	possible
Two drugs rated as possible (=2)		1	6	2	1	0	2	yes	possible
Two drugs rated as probable (=3)		1	1	4	3	1	3	no	probable
Serotonin syndrome									
SSRI		0	1	6	3	0	3	no	probable
SSNRI		0	1	7	2	0	3	no	probable
MAO inhibitors		0	1	7	1	1	3	no	probable
Tricyclic and tetracyclic antidepressants		0	6	3	1	0	2	yes	possible
5-HT2A antagonists		2	5	1	0	2	2	no	possible
Certain atypical antipsychotics	Risperidone, Clozapine, Olanzapine, Quetiapine, Amisulpride, Aripiprazole	3	6	1	0	0	2	no	possible
Triptans		5	4	0	1	0	1.5	no	unlikely

Certain antiemetics	Ondansetron, Granisetron, Metoclopramide	3	7	0	0	0	2	no	possible
Certain opioids	Tramadol, Fentanyl, Methadone, Tapentadol, Buprenorphine, Pethidine, Oxycodone, Codeine, Dextromethorphan	1	7	1	1	0	2	no	possible
Certain antibiotics with MAO-inhibiting activity	Linezolid, Tedizolid, Isoniazid	2	3	4	1	0	2.5	yes	possible
Amphetamines and derivatives		4	4	0	0	2	1.5	no	unlikely
Certain antiepileptics	Carbamazepine, Oxcarbazepine, Valproic acid	5	4	0	1	0	1.5	no	unlikely
Miscellaneous drugs	Bupropion, Buspirone, Chlorphenamine, Lithium, Procarbazine, St John's wort, Thioridazine	3	5	1	0	1	2	no	possible
Two drugs rated as possible (=2)		1	1	8	0	0	3	no	probable
Two drugs rated as probable (=3)		0	0	6	4	0	3	no	probable
Agranulocytosis / Neutropenia									
Cytotoxic anticancer drugs		0	0	7	3	0	3	no	probable
Certain antivirals	*Antiretroviral therapy:* Indinavir, Abacavir, Zidovudine<br>*Other:* Acyclovir, Valacyclovir, Oseltamivir, Ganciclovir, Valganciclovir	1	3	6	0	0	3	yes	possible
ACE inhibitors		8	2	0	0	0	1	no	unlikely
Antiplatelet drugs (excl. ASA)		7	3	0	0	0	1	no	unlikely
Vitamin K antagonists		10	0	0	0	0	1	no	unlikely
Certain antiarrhythmics (excl. Cardiac glycosides)	Class Ia: Ajmaline<br>Class Ib: Tocainide<br>Class Ic: Flecainide, Propafenone<br>Class III: Amiodarone	7	1	1	0	1	1	no	unlikely
Cardiac glycosides		8	2	0	0	0	1	no	unlikely
Thiazides		6	4	0	0	0	1	no	unlikely

Certain anticonvulsants	Phenobarbital, Carbamazepine, Ethosuximide, Lamotrigine, Levetiracetam, Phenytoin, Valproate	3	4	3	0	0	2	yes	possible
Certain H1-receptor blockers (first generation)	Chlorpheniramine, Tripelenamine	5	2	0	0	3	1	no	unlikely
H2-receptor blockers		6	4	0	0	0	1	no	unlikely
Phenothiazines		4	6	0	0	0	2	no	possible
Clozapine		0	4	3	3	0	3	yes	possible
Certain other neuroleptics (excl. Clozapine and Phenothiazines)	Olanzapine, Quetiapine, Ziprasidone, Haloperidol, Tiapride, Risperidone	3	6	1	0	0	2	no	possible
Retinoids		3	7	0	0	0	2	no	possible
Tricyclic and tetracyclic antidepressants		5	5	0	0	0	1.5	no	unlikely
NSAIDs (excl. Pyrazolones)		7	3	0	0	0	1	no	unlikely
Pyrazolones		0	2	6	2	0	3	no	probable
Beta-lactams		3	6	1	0	0	2	no	possible
Macrolides		7	3	0	0	0	1	no	unlikely
Tetracyclines		6	3	0	0	1	1	no	unlikely
Sulphonamides		2	4	4	0	0	2	yes	possible
Certain other antibiotics	*Lincosamide:* Clindamycin, Lincomycin<br>*Glycopeptide antibiotics:* Teicoplanin, Vancomycin<br>*Fluoroquinolones:* Norfloxacin, Ciprofloxacin<br>*Aminoglycosides:* Streptomycin, Gentamycin, Tobramycin<br>*Antituberculotics:* Rifampicin, Isoniazid, Ethambutol<br>*Other:* Fusidic acid, Chlorampenicol, Nifuroxazid, Nitrofurantoin, Metonidazole, Linezolid, Dapsone	3	5	1	0	1	2	no	possible

Anti-malaria drugs		4	3	2	0	1	2	no	possible
Certain antimycotics	Ketoconazole, Fluconazole, Amphotericin B, 5-Flucytosine, Terbinafine	5	4	1	0	0	1.5	no	unlikely
Sulfonylureas		7	2	1	0	0	1	no	unlikely
Glucocorticoids		9	1	0	0	0	1	no	unlikely
Thionamides		1	7	2	0	0	2	no	possible
Certain immunosuppressants (excl. Biologicals)	*mTor inhibitors:* Sirolimus, Everolimus<br><br>*Other:* Tacrolimus, Lenalidomide, Tofacitinib, Leflunomide, Azathioprine, MMF	0	5	5	0	0	2.5	yes	possible
Biologicals		1	6	3	0	0	2	yes	possible
Miscellaneous drugs	Acetylcysteine, Allopurinol, Bezafibrate, Chloralhydrat, Chlordiazepoxide, Colchicine, Deferiprone, Diazepam, Diflunisal, Fluoxetine, Flutamide, Furosemide, Hydralazine, Imatinib, Levodopa, Mebendazole, Methyldopa, Metoclopramide, Mesalazine, Nifedipine, Omeprazole, Paracetamol, Penicillamine, Propranolol, Riluzole, Spironolactone, Tamoxifen, Venlafaxine	4	5	0	0	1	2	no	possible
Two drugs rated as possible (=2)		1	5	4	0	0	2	yes	possible
Two drugs rated as probable (=3)		1	1	6	1	1	3	no	probable
Acute kidney injury									
NSAIDs		0	3	6	1	0	3	yes	possible
Anti-angiogenesis drugs		1	7	1	0	1	2	no	possible
Aminoglycosides		0	0	7	3	0	3	no	probable
Beta-lactams		5	4	1	0	0	1.5	no	unlikely

Fluoroquinolones/Quinolones		3	6	1	0	0	2	no	possible
Macrolides		6	4	0	0	0	1	no	unlikely
Polymyxins		0	4	4	1	1	3	yes	possible
Sulphonamides		3	7	0	0	0	2	no	possible
Tetracylines		5	5	0	0	0	1.5	no	unlikely
Certain other antibiotics	Clindamycin, Chloramphenicol, Ethambutol, Nitrofurantoin, Rifampicin, Vancomycin	2	3	4	0	1	2	yes	possible
Amphotericin B		0	3	5	2	0	3	yes	possible
Certain antiepileptic drugs	Carbamazepine, Phenobarbital, Phenytoin, Topiramate, Valproate, Zonisamide	5	5	0	0	0	1.5	no	unlikely
ARBs		2	6	2	0	0	2	no	possible
ACE inhibitors		1	6	3	0	0	2	yes	possible
Certain antineoplastic agents	*Platin compounds:* Carboplatin, Cisplatin, Oxaliplatin<br>*Checkpoint inhibitors:* Ipilimumab, Nivolumab, Pembrolizumab<br>*Antimetabolites:* Gemcitabine, Pemetrexed<br>*Other:* Mitomycin C, Doxorubicin, Methotrexate, Ifosfamide	0	3	6	0	1	3	yes	possible
Bisphosphonates		4	5	1	0	0	2	no	possible
Certain antiplatelet drugs	Clopidogrel, Ticlopidine	10	0	0	0	0	1	no	unlikely
Certain antivirals	*Protease inhibitors:* Indinavir, Atazanavir<br>*NRTIs*: Abacavir, Adefovir, Tenofovir<br>*Nucleoside analogues:* Acyclovir, Ganciclovir, Valacyclovir, Valganciclovir<br>*Other:* Cidofovir, Foscarnet	0	6	3	0	1	2	yes	possible
Contraceptive agents		9	0	1	0	0	1	no	unlikely
Contrast agents (i.v.)		0	2	8	0	0	3	no	probable

Thiazides		4	6	0	0	0	2	no	possible
Loop diuretics		4	4	2	0	0	2	no	possible
Potassium-sparing diuretics		6	4	0	0	0	1	no	unlikely
Certain H2-receptor blockers	Cimetidine, Ranitidine	9	1	0	0	0	1	no	unlikely
Proton pump inhibitors		5	4	1	0	0	1.5	no	unlikely
Calcineurin inhibitors / mTor inhibitors		0	3	7	0	0	3	yes	possible
5-Aminosalicylates		2	7	0	0	1	2	no	possible
Rhabdomyolysis-inducing drugs		3	4	3	0	0	2	yes	possible
Miscellaneous drugs	Allopurinol, Deferasirox, Ephedrine, Guaifenesin, Hydralazine, Infliximab, Interferons, Intravenous human globulins, Lithium, Paracetamol, Penicillamine, Pentamidine, Propylthiouracil, Quinine, Warfarin	1	8	0	0	1	2	no	possible
Two drugs rated as possible (=2)		0	5	5	0	0	2.5	yes	possible
Two drugs rated as probable (=3)		0	1	5	4	0	3	no	probable
Rhabdomyolysis									
Certain antibiotics	*Fluoroquinolones:* Levofloxacin, Ofloxacin *Macrolides:* Erythromycin, Clarithromycin *Other:* Daptomycin, Cotrimoxazole, Penicillin-Benzathine, Isoniazid, Pyrazinamide	3	6	0	0	1	2	no	possible
Certain antimycotics	Amphotericin B, Fluconazole, Itraconazole (+ Statin), Ketoconazole (+Statin), Voriconazole, Posaconazole, Terbinafine	3	6	1	0	0	2	no	possible
Certain antivirals	Tenofovir, Ritonavir, Ganciclovir, Letermovir, Simeprevir, Etravirin, Didanosine, Darunavir, Atazanavir, Tipranavir, Saquinavir, Raltegravir, Fosamprenavir, Indinavir, Lamivudine, Maraviroc, Nevirapin, Zidovudine	1	8	0	0	1	2	no	possible

Certain antihistamines	Diphenhydramine, Doxylamine, Cimetidine, Famotidine, Hydroxyzine	7	3	0	0	0	1	no	unlikely
Antipsychotics (typical/atypical)		2	7	0	0	1	2	no	possible
Antidepressants		3	6	0	0	1	2	no	possible
Statins		0	0	5	5	0	3.5	no	probable
Fibrates		0	4	4	2	0	3	yes	possible
Certain cytostatic drugs	Cytarabine, Nelarabine, Azacytidine, Oxaliplatin, Cyclophosphamide (+ Mitoxantrone), Ifosfamide, Trabectedin	2	8	0	0	0	2	no	possible
Certain hypnotics	*Barbiturates:* Phenobarbital<br>*Benzodiazepines:* Diazepam, Lorazepam, Nitrazepam, Flunitrazepam, Triazolam	4	5	0	0	1	2	no	possible
Certain anticonvulsants	Phenytoin, Felbamate, Lamotrigine, Zonisamide, Pregabalin, Gabapentin	6	4	0	0	0	1	no	unlikely
Thiazides		9	1	0	0	0	1	no	unlikely
Opioids		9	1	0	0	0	1	no	unlikely
NSAIDs		10	0	0	0	0	1	no	unlikely
Corticosteroids		8	1	0	0	1	1	no	unlikely
Retinoids		5	3	0	0	2	1	no	unlikely
Certain iodinated contrast media	Iodixanol, Iohexol, Iopamidol, Iopromid, Ioversol	4	5	0	0	1	2	no	possible
Miscellaneous drugs with most concern according to DIRA	Certain drugs with most concern according to DIRA: Alteplase, Baclofen, Ciclosporin, Interferon-alfa-2b, Nivolumab, Succinylcholine, Sunitinib, Tolcapone, Ziconotide	2	5	2	0	1	2	no	possible
Miscellaneous drugs with possible concern according to DIRA	Certain drugs with possible concern according to DIRA: Aldesleukin, Diltiazem, Quinine, Cobimetinib, Colchicine, Everolimus, Lithium, Rotigotine, Sirolimus, Vasopressin, Verapamil	3	6	1	0	0	2	no	possible
Miscellaneous drugs with less concern according to DIRA	Certain drugs with less concern according to DIRA: Abirateronacetate, Amiodarone,	6	4	0	0	0	1	no	unlikely

	Dasatinib, Desflurane, Donezepil, Entacapone, Erlotinib, Ezetimibe, Febuxostat, Imatinib, Losartan, Olmesartan, Peginterferon alfa-2b, Propofol, Sonidegib, Sorafenib Sulfasalazine, Tacrolimus, Temsirolimus, Theophyllline, Trametinib								
Miscellaneous drugs not listed in DIRA	Certain drugs not listed in DIRA: Azathioprine, Leflunomide, Paracetamol	8	1	0	0	1	1	no	unlikely
Two drugs rated as possible (=2)		0	7	3	0	0	2	yes	possible
Two drugs rated as probable (=3)		0	1	6	3	0	3	no	probable
Delirium									
Total ACB-Score: 1 point		5	5	0	0	0	1.5	no	unlikely
Total ACB-Score: 2 points		4	6	0	0	0	2	no	possible
Total ACB-Score: ≥ 3 points		0	3	6	1	0	3	yes	possible
SSRI		1	9	0	0	0	2	no	possible
Anticonvulsants (excl. Phenobarbitals)		1	8	0	0	1	2	no	possible
Dopamine agonists		1	6	1	0	2	2	no	possible
Narcotics		0	3	5	1	1	3	yes	possible
GABA-receptor agonists		0	7	3	0	0	2	yes	possible
Antiarrhythmics (excl. Digitalis glycosides and Beta-blocking agents)		8	2	0	0	0	1	no	unlikely
Digitalis glycosides		5	4	1	0	0	1.5	no	unlikely
Beta-blocking agents		8	2	0	0	0	1	no	unlikely
Certain antibiotics	Beta-lactams/Cephalosporins, Quinolone/Fluoroquinolone, Macrolides, Antituberculotics	6	2	1	0	1	1	no	unlikely

Diuretics		5	3	2	0	0	1.5	no	unlikely
Antidiabetics		5	4	1	0	0	1.5	no	unlikely
Glucocorticoids (systemic)		6	2	2	0	0	1	no	unlikely
NSAIDs		7	3	0	0	0	1	no	unlikely
Opiates		2	4	4	0	0	2	yes	possible
Miscellaneous drugs	Bupropion, Disulfiram, Interferon Lithium, Methyldopa, Theophylline	2	6	1	0	1	2	no	possible
Two drugs rated as possible (=2)		0	4	6	0	0	3	yes	possible
Two drugs rated as probable (=3)		0	0	7	2	1	3	no	probable
Liver damage									
1 drug from category A according to LiverTox®		0	2	6	2	0	3	no	probable
1 drug from category B according to LiverTox®		0	5	5	0	0	2.5	yes	possible
1 drug from category C according to LiverTox®		0	10	0	0	0	2	no	possible
1 drug from category D according to LiverTox®		3	6	0	0	1	2	no	possible
1 drug from category E according to LiverTox®		8	1	0	0	1	1	no	unlikely
1 drug from category E* according to LiverTox®		7	3	0	0	0	1	no	unlikely
Two drugs rated as possible (=2)		0	5	5	0	0	2.5	yes	possible
Two drugs rated as probable (=3)		0	1	6	3	0	3	no	probable
Torsade de pointes tachycardia									
1 drug with known risk of TdP according to CredibleMeds®		0	4	4	2	0	3	yes	possible

2 drugs taken simultaneously with known risk of TdP according to CredibleMeds®		0	3	4	3	0	3	yes	possible
1 drug with possible risk of TdP according to CredibleMeds®		1	8	1	0	0	2	no	possible
2 drugs taken simultaneously with possible risk of TdP according to CredibleMeds®		0	6	4	0	0	2	yes	possible
2 drugs taken simultaneously with conditional risk of TdP according to CredibleMeds®		1	9	0	0	0	2	no	possible
1 drug with known risk of TdP + 1 drug with possible risk of TdP according to CredibleMeds® (taken simultaneously)		0	4	6	0	0	3	yes	possible
1 drug with known risk of TdP + 1 drug with conditional risk of TdP according to CredibleMeds® (taken simultaneously)		0	6	4	0	0	2	yes	possible
1 drug with possible risk of TdP + 1 drug with conditional risk of TdP according to CredibleMeds® (taken simultaneously)		1	7	2	0	0	2	no	possible

Abbreviations: 5-HT2A: 5-hydroxytryptamine 2A receptor; 5-HT3: 5-hydroxytryptamine 3 receptor; ACB score: Anticholinergic burden score by Kiesel et al.; ACE: Angiotensin-converting enzyme; ARBs: Angiotensin receptor blockers; ASA: Acetylsalicylic acid; CCBs: Calcium channel blockers; COX: Cyclooxygenase; DIRA: Drug-induced rhabdomyolysis atlas; ENaC: Epithelial sodium channel; GIT: Gastrointestinal tract; Excl.: Exclusive; MAO: Monoamine oxidase; MMF: Mycophenolate mofetil; mTOR: Mammalian target of rapamycin; NRTIs: Nucleoside/nucleotide reverse transcriptase inhibitors; NSAIDs: Non-steroidal anti-inflammatory drugs; SSNRI: Selective serotonin and norepinephrine reuptake inhibitors; SSRI: Selective serotonin reuptake inhibitors; TdP: Torsade de pointes tachycardia; TKIs: Tyrosine kinase inhibitors

Table S5-2 Expert ratings of the second round

		Number of participants with rating							
Drug-event pairs sorted by event	Certain drugs	1	2	3	4	0	Median	Disagreement	Category
Hyperkalemia									
ACE inhibitors		0	6	3	0	0	2	yes	possible
ARBs		0	7	2	0	0	2	no	possible
Direct renin inhibitors		0	7	2	0	0	2	no	possible
Aldosterone antagonists		0	3	6	0	0	3	yes	possible
ENaC blockers		1	4	4	0	0	2	yes	possible
Beta-blocking agents		7	1	1	0	0	1	no	unlikely
CCBs		8	1	0	0	0	1	no	unlikely
NSAIDs		2	6	1	0	0	2	no	possible
Heparin and derivatives		4	5	0	0	0	2	no	possible
Tacrolimus		0	7	2	0	0	2	no	possible
Other calcineurin inhibitors (excl. Tacrolimus)		2	6	1	0	0	2	no	possible
Pentamidine		2	4	2	0	1	2	no	possible
Cotrimoxazole		1	3	4	0	1	2.5	yes	possible
Agents containing a high amount of potassium		0	1	6	2	0	3	no	probable
Miscellaneous drugs	Digoxin, Mannitol, Propofol, Ketoconazole, Drospirenone, Magnesium sulphate	4	5	0	0	0	2	no	possible
Suxamethonium		0	3	5	1	0	3	yes	possible

Two drugs rated as possible (=2)		0	2	7	0	0	3	no	probable
Two drugs rated as probable (=3)		0	0	7	1	1	3	no	probable
Hyponatremia									
Oncologicals (excl. TKIs)		1	6	1	0	1	2	no	possible
TKIs		3	5	0	0	1	2	no	possible
MAO inhibitors		3	4	1	0	1	2	no	possible
SSNRI / SSRI		0	2	7	0	0	3	no	probable
Tricyclic and tetracyclic antidepressants		0	6	3	0	0	2	yes	possible
Antipsychotics		1	6	2	0	0	2	no	possible
Thiazides		0	1	7	1	0	3	no	probable
Other diuretics		0	2	7	0	0	3	no	probable
ACE inhibitors		4	4	1	0	0	2	no	possible
Carbamazepine and analogues		0	4	3	2	0	3	yes	possible
Other anticonvulsants (excl. Carbamazepine and analogues)		3	4	2	0	0	2	no	possible
Vasopressin and analogues		0	2	5	1	1	3	no	probable
Certain laxatives	Macrogol, Sodium picosulfate	2	7	0	0	0	2	no	possible
NSAIDs		8	0	1	0	0	1	no	unlikely
Opiates		7	1	1	0	0	1	no	unlikely
Proton pump inhibitors		7	2	0	0	0	1	no	unlikely
Certain antibiotics	Ciprofloxacin, Linezolid, Rifabutin, Sulfamethoxazole/Trimethoprim	8	1	0	0	0	1	no	unlikely

Miscellaneous drugs	Amiodarone, Amlodipine, Bromocriptine, Bupropion, Immunoglobulin, Paracetamol, Propafenone, Theophylline, Tolbutamide, Voriconazole	7	2	0	0	0	1	no	unlikely
Two drugs rated as possible (=2)		0	4	5	0	0	3	yes	possible
Two drugs rated as probable (=3)		0	1	6	2	0	3	no	probable
Hypoglycemia									
Incretin therapy		4	4	1	0	0	2	no	possible
Sulfonylureas		0	1	3	5	0	4	no	certain
Glinides		0	4	4	1	0	3	yes	possible
Non-insulinotropic antidiabetics		5	3	1	0	0	1	no	unlikely
Insulin		0	0	3	6	0	4	no	certain
ACE inhibitors		7	1	1	0	0	1	no	unlikely
Beta-blocking agents		4	5	0	0	0	2	no	possible
Certain ARBs	Losartan, Telmisartan, Valsartan	8	1	0	0	0	1	no	unlikely
Certain α2-receptor agonists	Clonidine, Dexmedetomidine	8	1	0	0	0	1	no	unlikely
Certain CCBs (Dihydropyridine type)	Nifedipine, Felodipine	9	0	0	0	0	1	no	unlikely
Certain serotonergic antidepressants	Venlafaxine, Sertraline, Fluoxetine, Imipramine, Maprotiline, Nortriptyline, Doxepin	9	0	0	0	0	1	no	unlikely
Certain anticonvulsants	Gabapentin, Phenytoin, Topiramate, Valproate	7	2	0	0	0	1	no	unlikely
Anti-malaria drugs		3	4	0	0	2	2	no	possible
Quinolones		5	4	0	0	0	1	no	unlikely
Certain other antibiotics	Ceftriaxone, Clarithromycin, Doxycycline, Tetracycline, Oxytetracycline, Isoniazid, Para-aminosalicylic acid, Pentamidine, Piperacillin-	7	2	0	0	0	1	no	unlikely

	tazobactam, Sulfadiazine, Sulfamethoxazole/ Trimethoprim								
Azole antimycotics		8	1	0	0	0	1	no	unlikely
Certain NSAIDs	Ibuprofen, Indomethacin, Phenylbutazone, Salicylates, Piroxicam	5	4	0	0	0	1	no	unlikely
Certain antivirals	Amprenavir, Entecavir, Ganciclovir, Saquinavir, Stavudine, Zidovudine	6	3	0	0	0	1	no	unlikely
Certain growth hormones	IGF-I (Mecasermin), Somatotropin	3	4	1	0	1	2	no	possible
Miscellaneous drugs	Amiodarone, Atorvastatin, Bortezomib, Chlormadinone, Clonazepam Propoxyphene/Dextropropoxyphene, Donepezil, Imatinib, Haloperidol, Etanercept, Ethacrynic acid, Etomidate, Hydralazine, Lidocaine, IL-2, Interferons, Lenalidomide, Lithium, Levothyroxine, 6-Mercaptopurine, Mifepristone, Octreotide, Paracetamol, Ranitidine, Salbutamol, Selegiline, Tramadol	8	0	0	0	1	1	no	unlikely
Two drugs rated as possible (=2)		0	6	3	0	0	2	yes	possible
Two drugs rated as probable (=3)		0	0	7	2	0	3	no	probable
Bleeding of the upper GIT									
NSAIDs (non-selective)		0	0	8	1	0	3	no	probable
Selective COX-2 inhibitors		1	6	2	0	0	2	no	possible
Paracetamol		6	3	0	0	0	1	no	unlikely
Direct oral anticoagulants		0	1	6	2	0	3	no	probable
Vitamin K antagonists		0	1	6	2	0	3	no	probable
Antiplatelet drugs		0	1	6	2	0	3	no	probable
CCBs		9	0	0	0	0	1	no	unlikely

SSRI		1	6	2	0	0	2	no	possible
Glucocorticoids		1	6	2	0	0	2	no	possible
Certain angiogenesis inhibitors	Bevacizumab, Erlotinib, Axitinib, Aflibercept	1	2	5	0	1	3	yes	possible
Two drugs rated as possible (=2)		0	5	3	1	0	2	yes	possible
Two drugs rated as probable (=3)		0	0	7	2	0	3	no	probable
Bleeding outside the GIT									
NSAIDs (non-selective; excl. Diclofenac and ASA)		2	5	2	0	0	2	no	possible
ASA		0	1	8	0	0	3	no	probable
Diclofenac		2	5	2	0	0	2	no	possible
Heparins		0	2	6	1	0	3	no	probable
Vitamin K antagonists		0	1	6	2	0	3	no	probable
Direct oral anticoagulants		0	1	6	2	0	3	no	probable
Certain other anticoagulants	Fondaparinux, Argatroban, Bivalirudin	0	2	4	2	1	3	no	probable
Other antiplatelet drugs (excl. Dipyridamole, Cilostazol, ASA)		0	2	6	1	0	3	no	probable
Certain phosphodiesterase inhibitors	Dipyridamole, Cilostazol	1	4	2	0	2	2	no	possible
SSRI / SSNRI		2	7	0	0	0	2	no	possible
Certain angiogenesis inhibitors	Ranibizumab, Bevacizumab, Sorafenib	1	5	2	0	1	2	no	possible
Certain anticancer drugs	Fludarabine, Cytarabine, Cyclophosphamide, Ifosfamide, Oxaliplatin, Ramucirumab, Ibrutinib, Acalabrutinib, Zanubrutinib, Gefitinib, Azathioprine/Mercaptopurine	0	6	3	0	0	2	yes	possible
Certain beta-lactams	*Penicillin's:* Penicillin, Ampicillin, Piperacillin *Cephalosporins:* Ceftriaxone	6	2	0	0	1	1	no	unlikely

Certain other antibiotics	Trimethoprim/Sulfamethoxazole, Vancomycin, Rifampicin, Mitomycin, Nitrofurantoine	6	2	0	0	1	1	no	unlikely
Certain antiepileptics	Phenytoin, Carbamazepine, Valproic acid	6	2	0	0	1	1	no	unlikely
Fibrinolytics		0	0	7	2	0	3	no	probable
Miscellaneous drugs	Amlodipine, Mirtazapine, Quinine	8	0	0	0	1	1	no	unlikely
Two drugs rated as possible (=2)		0	3	6	0	0	3	yes	possible
Two drugs rated as probable (=3)		0	0	7	2	0	3	no	probable
Stevens-Johnson syndrome and toxic epidermal necrolysis									
Certain antiepileptics	Carbamazepine, Phenytoin, Phenobarbital, Valproate, Lamotrigine, Oxcarbazepine, Levetiracetam	0	3	5	1	0	3	yes	possible
Antibiotics (excl. Sulphonamides and Antituberculotics)		0	6	3	0	0	2	yes	possible
Sulphonamides		0	3	6	0	0	3	yes	possible
Antituberculotics		1	7	1	0	0	2	no	possible
Nevirapine		0	3	5	0	1	3	yes	possible
Antiretroviral therapy		2	4	2	0	1	2	no	possible
Certain antimycotics	Fluconazole, Nystatin, Terbinafine	3	4	2	0	0	2	no	possible
NSAIDs + Paracetamol		0	8	1	0	0	2	no	possible
Certain diuretics	Furosemide, Acetazolamide	3	6	0	0	0	2	no	possible
Glucocorticoids		4	5	0	0	0	2	no	possible
Allopurinol		0	3	6	0	0	3	yes	possible
Certain antidepressants	Sertraline, Mirtazapine, Duloxetine	0	8	1	0	0	2	no	possible
Certain anticancer drugs	*Taxanes:* Docetaxel, Paclitaxel	2	7	0	0	0	2	no	possible

	TKIs: Afatinib, Imatinib, Sunitinib, Vandetanib, *Checkpoint inhibitors:* Ipilimumab, Nivolumab, Pembrolizumab *Other:* Cetuximab, Leflunomide, Lenalidomide, Tamoxifen, Thalidomide, Vemurafenib								
Miscellaneous drugs	Amlodipine, Chloroquine, Famotidine, Isotretinoin, Oseltamivir, Pantoprazole, Ramipril, Tramadol	1	7	0	0	1	2	no	possible
Two drugs rated as possible (=2)		0	7	1	1	0	2	no	possible
Two drugs rated as probable (=3)		0	3	3	2	1	3	yes	possible
Anaphylaxis									
Beta-lactams		0	1	6	2	0	3	no	probable
Fluoroquinolones		1	6	1	1	0	2	no	possible
Sulphonamides		0	6	2	1	0	2	yes	possible
Macrolides		2	6	0	1	0	2	no	possible
Tetracyclines		0	7	0	1	1	2	no	possible
Vancomycin		1	1	5	2	0	3	no	probable
Glycopeptide antibiotics (excl. Vancomycin)		2	5	2	0	0	2	no	possible
Certain other antibiotics	Clindamycin, Metronidazole, Rifampicin, Bacitracin, Gentamicin, Tobramycin	3	5	1	0	0	2	no	possible
Anaesthetic agents		2	6	1	0	0	2	no	possible
Local anaesthetics		1	4	4	0	0	2	yes	possible
Glucocorticoids		7	2	0	0	0	1	no	unlikely
Certain chemotherapy agents (excl. Taxanes)	*Platinum compounds:* Carboplatin, Cisplatin, Oxaliplatin *Topoisomerase II inhibitors:* Podophyllotoxins (Etoposide, Teniposid), Doxorubicin	2	5	2	0	0	2	no	possible

	Other: L-Asparaginase, Procarbazine, Cyclophosphamide, Cytarabine, Methotrexate, Leflunomide								
Taxanes		1	2	5	1	0	3	yes	possible
Iodinated contrast media		0	1	7	1	0	3	no	probable
Other contrast media		0	7	2	0	0	2	no	possible
Neuromuscular blocking agents		0	3	5	0	1	3	yes	possible
NSAIDs		2	5	2	0	0	2	no	possible
Opioids		1	8	0	0	0	2	no	possible
Paracetamol		4	4	1	0	0	2	no	possible
Metamizole (i.v.)		0	4	5	0	0	3	yes	possible
Proton pump inhibitors		6	2	1	0	0	1	no	unlikely
Certain 5-HT3 antagonists	Ondansetron, Palonosetron	5	4	0	0	0	1	no	unlikely
Certain uterotonic drugs	Oxytocin, Dinoprostone	4	4	0	0	1	1.5	no	unlikely
Biologicals with immunological target		0	2	3	2	2	3	no	probable
Biologicals without immunological target		1	2	3	1	2	3	yes	possible
Miscellaneous drugs	Abacavir, Atropine, Cetirizine, Heparin, Hyaluronidase, Mannitol, Neostigmine, Sugammadex, Tranexamic acid	2	6	0	0	1	2	no	possible
Protamine		0	7	1	1	0	2	no	possible
Two drugs rated as possible (=2)		1	5	2	1	0	2	yes	possible
Two drugs rated as probable (=3)		1	0	5	2	1	3	no	probable
Serotonin syndrome									

SSRI		0	0	6	3	0	3	no	probable
SSNRI		0	0	7	2	0	3	no	probable
MAO inhibitors		0	1	7	1	0	3	no	probable
Tricyclic antidepressants (excl. Clomipramine/ Imipramine)		0	5	3	1	0	2	yes	possible
Clomipramine and Imipramine		0	5	3	1	0	2	yes	possible
Tetracyclic antidepressants		2	5	1	1	0	2	no	possible
5-HT2A antagonists		3	4	1	0	1	2	no	possible
Certain atypical antipsychotics	Risperidone, Clozapine, Olanzapine, Quetiapine, Amisulpride, Aripiprazole	4	4	1	0	0	2	no	possible
Triptans		4	4	0	1	0	2	no	possible
Certain antiemetics	Ondansetron, Granisetron, Metoclopramide	3	6	0	0	0	2	no	possible
Certain opioids	Tramadol, Fentanyl, Methadone, Tapentadol, Buprenorphine, Pethidine, Oxycodone, Codeine, Dextromethorphan	0	7	1	1	0	2	no	possible
Certain antibiotics with MAO-inhibiting activity	Linezolid, Tedizolid, Isoniazid	1	3	4	1	0	3	yes	possible
Amphetamines and derivatives		3	4	0	0	2	2	no	possible
Certain antiepileptics	Carbamazepine, Oxcarbazepine, Valproic acid	5	3	0	1	0	1	no	unlikely
Miscellaneous drugs	Bupropion, Buspirone, Chlorphenamine, Lithium, Procarbazine, St John's wort, Thioridazine	2	5	1	0	1	2	no	possible
Two drugs rated as possible (=2)		0	1	8	0	0	3	no	probable
Two drugs rated as probable (=3)		0	0	6	3	0	3	no	probable
Agranulocytosis / Neutropenia									
Cytotoxic anticancer drugs		0	0	7	2	0	3	no	probable

Certain antivirals (excl. Ganciclovir and Valganciclovir)	*Antiretroviral therapy:* Indinavir, Abacavir, Zidovudine<br>*Other:* Acyclovir, Valacyclovir, Oseltamivir	2	6	1	0	0	2	no	possible
Ganciclovir and Valganciclovir		0	3	6	0	0	3	yes	possible
ACE inhibitors		7	2	0	0	0	1	no	unlikely
Antiplatelet drugs (excl. ASA)		7	2	0	0	0	1	no	unlikely
Vitamin K antagonists		9	0	0	0	0	1	no	unlikely
Certain antiarrhythmics (excl. Cardiac glycosides)	Class Ia: Ajmaline<br>Class Ib: Tocainide<br>Class Ic: Flecainide, Propafenone<br>Class III: Amiodarone	7	0	1	0	1	1	no	unlikely
Cardiac glycosides		8	1	0	0	0	1	no	unlikely
Thiazides		5	4	0	0	0	1	no	unlikely
Certain anticonvulsants	Phenobarbital, Carbamazepine, Ethosuximide, Lamotrigine, Levetiracetam, Phenytoin, Valproate	3	4	2	0	0	2	no	possible
Certain H1-receptor blockers (first generation)	Chlorpheniramine, Tripelenamine	4	4	0	0	1	1.5	no	unlikely
H2-receptor blockers		5	4	0	0	0	1	no	unlikely
Phenothiazines		3	6	0	0	0	2	no	possible
Clozapine		0	1	5	3	0	3	no	probable
Certain other neuroleptics (excl. Clozapine and Phenothiazines)	Olanzapine, Quetiapine, Ziprasidone, Haloperidol, Tiapride, Risperidone	3	5	1	0	0	2	no	possible
Retinoids		2	7	0	0	0	2	no	possible
Tricyclic and tetracyclic antidepressants		5	4	0	0	0	1	no	unlikely
NSAIDs (excl. Pyrazolones)		6	3	0	0	0	1	no	unlikely
Pyrazolones		0	2	5	2	0	3	no	probable

Beta-lactams		3	5	1	0	0	2	no	possible
Macrolides		6	3	0	0	0	1	no	unlikely
Tetracyclines		5	3	0	0	1	1	no	unlikely
Sulphonamides		2	4	3	0	0	2	yes	possible
Certain other antibiotics	*Lincosamide:* Clindamycin, Lincomycin<br>*Glycopeptide antibiotics:* Teicoplanin, Vancomycin<br>*Fluoroquinolones:* Norfloxacin, Ciprofloxacin<br>*Aminoglycosides:* Streptomycin, Gentamycin, Tobramycin<br>*Antituberculotics:* Rifampicin, Isoniazid, Ethambutol<br>*Other:* Fusidic acid, Chlorampenicol, Nifuroxazid, Nitrofurantoin, Metonidazole, Linezolid, Dapsone	3	4	1	0	1	2	no	possible
Anti-malaria drugs		4	2	2	0	1	1.5	no	unlikely
Certain antimycotics	Ketoconazole, Fluconazole, Amphotericin B, 5-Flucytosine, Terbinafine	5	3	1	0	0	1	no	unlikely
Sulfonylureas		6	2	1	0	0	1	no	unlikely
Glucocorticoids		8	1	0	0	0	1	no	unlikely
Thionamides		1	6	2	0	0	2	no	possible
Certain immunosuppressants (excl. Biologicals, Azathioprine, MMF)	*mTor inhibitors:* Sirolimus, Everolimus<br><br>*Other:* Tacrolimus, Lenalidomide, Tofacitinib, Leflunomide	0	8	1	0	0	2	no	possible
MMF and Azathioprine		0	2	7	0	0	3	no	probable
Biologicals		1	6	2	0	0	2	no	possible
Miscellaneous drugs	Acetylcysteine, Allopurinol, Bezafibrate, Chloralhydrat, Chlordiazepoxide, Colchicine, Deferiprone, Diazepam, Diflunisal, Fluoxetine,	4	4	0	0	1	1.5	no	unlikely

	Flutamide, Furosemide, Hydralazine, Imatinib, Levodopa, Mebendazole, Methyldopa, Metoclopramide, Mesalazine, Nifedipine, Omeprazole, Paracetamol, Penicillamine, Propranolol, Riluzole, Spironolactone, Tamoxifen, Venlafaxine								
Two drugs rated as possible (=2)		1	4	4	0	0	2	yes	possible
Two drugs rated as probable (=3)		1	0	6	1	1	3	no	probable
Acute kidney injury									
NSAIDs		0	2	7	0	0	3	no	probable
Anti-angiogenesis drugs		1	7	1	0	0	2	no	possible
Aminoglycosides		0	0	6	3	0	3	no	probable
Beta-lactams		4	4	1	0	0	2	no	possible
Fluoroquinolones/Quinolones		3	5	1	0	0	2	no	possible
Macrolides		6	3	0	0	0	1	no	unlikely
Polymyxins (i.v.)		0	4	4	1	0	3	yes	possible
Sulphonamides		2	7	0	0	0	2	no	possible
Tetracylines		4	5	0	0	0	2	no	possible
Certain other antibiotics (excl. Vancomycin)	Clindamycin, Chloramphenicol, Ethambutol, Nitrofurantoin, Rifampicin	2	4	2	0	1	2	no	possible
Vancomycin		0	1	8	0	0	3	no	probable
Amphotericin B		0	3	4	2	0	3	yes	possible
Certain antiepileptic drugs	Carbamazepine, Phenobarbital, Phenytoin, Topiramate, Valproate, Zonisamide	5	4	0	0	0	1	no	unlikely
ARBs		1	6	2	0	0	2	no	possible

ACE inhibitors		0	6	3	0	0	2	yes	possible
Certain other antineoplastic agents (excl. Methotrexate, Cisplatin, Ifosfamide)	*Platin compounds:* Carboplatin, Oxaliplatin<br>*Checkpoint inhibitors:* Ipilimumab, Nivolumab, Pembrolizumab<br>*Antimetabolites:* Gemcitabine, Pemetrexed<br>*Other:* Mitomycin C, Doxorubicin	1	3	4	0	1	2.5	yes	possible
Methotrexate, Cisplatin, Ifosfamide		0	1	8	0	0	3	no	probable
Bisphosphonates		3	5	1	0	0	2	no	possible
Certain antiplatelet drugs	Clopidogrel, Ticlopidine	9	0	0	0	0	1	no	unlikely
Certain antiretroviral therapy	*Protease inhibitors:* Indinavir, Atazanavir<br>*NRTIs:* Abacavir, Adefovir, Tenofovir	1	8	0	0	0	2	no	possible
Certain other antivirals	*Nucleoside analogues:* Acyclovir, Ganciclovir, Valacyclovir, Valganciclovir<br>*Other:* Cidofovir, Foscarnet	0	2	7	0	0	3	no	probable
Contraceptive agents		8	0	1	0	0	1	no	unlikely
Contrast agents (i.v.)		0	1	8	0	0	3	no	probable
Thiazides		3	6	0	0	0	2	no	possible
Loop diuretics		3	4	2	0	0	2	no	possible
Potassium-sparing diuretics		5	4	0	0	0	1	no	unlikely
Certain H2-receptor blockers	Cimetidine, Ranitidine	8	1	0	0	0	1	no	unlikely
Proton pump inhibitors		5	3	1	0	0	1	no	unlikely
mTor inhibitors		1	5	2	0	1	2	no	possible
Calcineurin inhibitors		0	2	7	0	0	3	no	probable
5-Aminosalicylates		2	6	0	0	1	2	no	possible
Miscellaneous drugs	Allopurinol, Deferasirox, Ephedrine, Guaifenesin, Hydralazine, Infliximab, Interferons, Intravenous	1	7	0	0	1	2	no	possible

	human globulins, Lithium, Paracetamol, Penicillamine, Pentamidine, Propylthiouracil, Quinine, Warfarin								
Two drugs rated as possible (=2)		0	4	5	0	0	3	yes	possible
Two drugs rated as probable (=3)		0	1	5	3	0	3	no	probable
Rhabdomyolysis									
Certain antibiotics	*Fluoroquinolones:* Levofloxacin, Ofloxacin *Macrolides:* Erythromycin, Clarithromycin *Other:* Daptomycin, Cotrimoxazole, Penicillin-Benzathine, Isoniazid, Pyrazinamide	3	5	0	0	1	2	no	possible
Certain antimycotics	Amphotericin B, Fluconazole, Itraconazole (+ Statin), Ketoconazole (+Statin), Voriconazole, Posaconazole, Terbinafine	3	5	1	0	0	2	no	possible
Certain antivirals	Tenofovir, Ritonavir, Ganciclovir, Letermovir, Simeprevir, Etravirin, Didanosine, Darunavir, Atazanavir, Tipranavir, Saquinavir, Raltegravir, Fosamprenavir, Indinavir, Lamivudine, Maraviroc, Nevirapin, Zidovudine	1	7	0	0	1	2	no	possible
Certain antihistamines	Diphenhydramine, Doxylamine, Cimetidine, Famotidine, Hydroxyzine	6	3	0	0	0	1	no	unlikely
Antipsychotics (typical/atypical)		2	6	0	0	1	2	no	possible
Antidepressants		3	5	0	0	1	2	no	possible
Statins		0	0	5	4	0	3	no	probable
Fibrates		0	4	3	2	0	3	yes	possible
Certain cytostatic drugs (excl. Trabectedin)	Cytarabine, Nelarabine, Azacytidine, Oxaliplatin, Cyclophosphamide (+ Mitoxantrone), Ifosfamide	2	6	1	0	0	2	no	possible
Trabectedin		0	0	8	0	1	3	no	probable
Certain hypnotics	*Barbiturates:* Phenobarbital *Benzodiazepines:* Diazepam, Lorazepam, Nitrazepam, Flunitrazepam, Triazolam	3	5	0	0	1	2	no	possible

Certain anticonvulsants	Phenytoin, Felbamate, Lamotrigine, Zonisamide, Pregabalin, Gabapentin	5	4	0	0	0	1	no	unlikely
Thiazides		8	1	0	0	0	1	no	unlikely
Opioids		9	0	0	0	0	1	no	unlikely
NSAIDs		9	0	0	0	0	1	no	unlikely
Corticosteroids		8	1	0	0	0	1	no	unlikely
Retinoids		5	3	0	0	1	1	no	unlikely
Certain iodinated contrast media	Iodixanol, Iohexol, Iopamidol, Iopromid, Ioversol	4	4	0	0	1	1.5	no	unlikely
Miscellaneous drugs with most concern according to DIRA	Certain drugs with most concern according to DIRA: Alteplase, Baclofen, Ciclosporin, Interferon-alfa-2b, Nivolumab, Succinylcholine, Sunitinib, Tolcapone, Ziconotide	2	5	1	0	1	2	no	possible
Miscellaneous drugs with possible concern according to DIRA	Certain drugs with possible concern according to DIRA: Aldesleukin, Diltiazem, Quinine, Cobimetinib, Colchicine, Everolimus, Lithium, Rotigotine, Sirolimus, Vasopressin, Verapamil	3	5	1	0	0	2	no	possible
Miscellaneous drugs with less concern according to DIRA	Certain drugs with less concern according to DIRA: Abirateronacetate, Amiodarone, Dasatinib, Desflurane, Donezepil, Entacapone, Erlotinib, Ezetimibe, Febuxostat, Imatinib, Losartan, Olmesartan, Peginterferon alfa-2b, Propofol, Sonidegib, Sorafenib Sulfasalazine, Tacrolimus, Temsirolimus, Theophyllline, Trametinib	6	3	0	0	0	1	no	unlikely
Miscellaneous drugs not listed in DIRA	Certain drugs not listed in DIRA: Azathioprine, Leflunomide, Paracetamol	7	1	0	0	1	1	no	unlikely
Two drugs rated as possible (=2)		0	7	2	0	0	2	no	possible
Two drugs rated as probable (=3)		0	1	6	2	0	3	no	probable
Delirium									
Total ACB-Score: 1 point		4	5	0	0	0	2	no	possible

Total ACB-Score: 2 points		3	6	0	0	0	2	no	possible
Total ACB-Score: ≥ 3 points		0	2	6	1	0	3	no	probable
SSRI (excl. Paroxetine)		4	5	0	0	0	2	no	possible
Anticonvulsants (excl. Phenobarbitals and Carbamazepine)		0	9	0	0	0	2	no	possible
Dopamine agonists		1	6	1	0	1	2	no	possible
Narcotics		0	2	5	1	1	3	no	probable
GABA-receptor agonists		0	8	1	0	0	2	no	possible
Antiarrhythmics (excl. Digitalis glycosides and Beta-blocking agents)		7	2	0	0	0	1	no	unlikely
Digitalis glycosides		5	3	1	0	0	1	no	unlikely
Beta-blocking agents		7	2	0	0	0	1	no	unlikely
Certain antibiotics	Beta-lactams/Cephalosporins, Quinolone/Fluoroquinolone, Macrolides, Antituberculotics	5	2	1	0	1	1	no	unlikely
Diuretics		5	2	2	0	0	1	no	unlikely
Glucocorticoids (systemic)		5	2	2	0	0	1	no	unlikely
NSAIDs		6	3	0	0	0	1	no	unlikely
Opiates		2	3	4	0	0	2	yes	possible
Miscellaneous drugs	Bupropion, Disulfiram, Interferon Lithium, Methyldopa, Theophylline	2	5	1	0	1	2	no	possible
Two drugs rated as possible (=2)		0	2	7	0	0	3	no	probable
Two drugs rated as probable (=3)		0	0	6	2	1	3	no	probable
Liver damage									

1 drug from category A according to LiverTox®		0	2	5	2	0	3	no	probable
1 drug from category B according to LiverTox®		0	5	4	0	0	2	yes	possible
1 drug from category C according to LiverTox®		0	9	0	0	0	2	no	possible
1 drug from category D according to LiverTox®		2	6	0	0	1	2	no	possible
1 drug from category E according to LiverTox®		7	1	0	0	1	1	no	unlikely
1 drug from category E* according to LiverTox®		6	3	0	0	0	1	no	unlikely
Two drugs rated as possible (=2)		0	5	4	0	0	2	yes	possible
Two drugs rated as probable (=3)		0	1	5	3	0	3	no	probable
Torsade de pointes tachycardia									
1 drug with known risk of TdP according to CredibleMeds®		0	2	5	2	0	3	no	probable
2 drugs taken simultaneously with known risk of TdP according to CredibleMeds®		0	2	5	2	0	3	no	probable
1 drug with possible risk of TdP according to CredibleMeds®		1	7	1	0	0	2	no	possible
2 drugs taken simultaneously with possible risk of TdP according to CredibleMeds®		0	6	3	0	0	2	yes	possible
2 drugs taken simultaneously with conditional risk of TdP according to CredibleMeds®		1	8	0	0	0	2	no	possible
1 drug with known risk of TdP + 1 drug with possible risk of TdP according to CredibleMeds® (taken simultaneously)		0	2	7	0	0	3	no	probable
1 drug with known risk of TdP + 1 drug with conditional risk of TdP according to CredibleMeds® (taken simultaneously)		0	4	5	0	0	3	yes	possible

1 drug with possible risk of TdP + 1 drug with conditional risk of TdP according to CredibleMeds® (taken simultaneously)		1	7	1	0	0	2	no	possible

Abbreviations: 5-HT2A: 5-hydroxytryptamine 2A receptor; 5-HT3: 5-hydroxytryptamine 3 receptor; ACB score: Anticholinergic burden score by Kiesel et al.; ACE: Angiotensin-converting enzyme; ARBs: Angiotensin receptor blockers; ASA: Acetylsalicylic acid; CCBs: Calcium channel blockers; COX: Cyclooxygenase; DIRA: Drug-induced rhabdomyolysis atlas; ENaC: Epithelial sodium channel; GIT: Gastrointestinal tract; Excl.: Exclusive; MAO: Monoamine oxidase; MMF: Mycophenolate mofetil; mTOR: Mammalian target of rapamycin; NRTIs: Nucleoside/nucleotide reverse transcriptase inhibitors; NSAIDs: Non-steroidal anti-inflammatory drugs; SSNRI: Selective serotonin and norepinephrine reuptake inhibitors; SSRI: Selective serotonin reuptake inhibitors; TdP: Torsade de pointes tachycardia; TKIs: Tyrosine kinase inhibitors
