# Supplementary material for: Drug‐Event Pairs as Indicators for the Detection of Adverse Drug Reactions during Hospitalization in Routinely Collected Electronic Data Sources
Source: Clin Pharmacol Ther. 2025 Mar 18;117(6):1811–9. doi: 10.1002/cpt.3635 (PMC12087692; doi:10.1002/cpt.3635)
Supplement: Supplementary file 6 — Data S6. [file CPT-117-1811-s005.pdf]

Journal: Clinical Pharmacology & Therapeutics

Drug-Event Pairs as Indicators for the Detection of Adverse Drug Reactions during Hospitalization in Routinely Collected Electronic Data Sources

SUPPLEMENT S6: Adjustments after the panel meeting

Anna Maria Wermund[1], Annette Haerdtlein[2], Wolfgang Fehrmann[1], Clara Weglage[2], Tobias Dreischulte[2] and Ulrich Jaehde[1]*

[1] Department of Clinical Pharmacy, Institute of Pharmacy, University of Bonn, Bonn, Germany
[2] Institute of General Practice and Family Medicine, LMU University Hospital, LMU Munich, Munich, Germany
*Corresponding author. E-mail: u.jaehde@uni-bonn.de

Table S6 Adjustments after the panel meeting

Initial drug class (round 1)	Adjusted drug class (round 2)
Hyperkalemia	
Calcineurin inhibitors (whole group)	Tacrolimus
	Other calcineurin inhibitors
Certain anti-infectives (Pentamidine, Cotrimoxazole)	Pentamidine
	Cotrimoxazole
Potassium-containing agents: Penicillin G, Potassium citrate, Potassium supplements (oral or i.v.), Salt substitutes	Agents containing a high amount of potassium: Potassium citrate, Potassium supplements (oral or i.v.; high dose)
Miscellaneous drugs	Miscellaneous drugs (excl. Suxamethonium)
	Suxamethonium
Hyponatremia	
Oncologicals (whole group)	Oncologicals (excl. Tyrosine kinase inhibitors)
	Tyrosine kinase inhibitors
Anticonvulsants (whole group)	Anticonvulsants (excl. Carbamazepine and analogues)
	Carbamazepine and analogues
Hypoglycemia	
Classic insulin secretagogues	Sulfonylureas
	Glinides
Bleeding outside the gastrointestinal tract	
NSAIDs (non-selective; whole group)	NSAIDs (excl. Diclofenac and ASA)
	ASA

Initial drug class (round 1)	Adjusted drug class (round 2)
	Diclofenac
/	Fibrinolytics (whole group)
Anaphylaxis	
Glycopeptide antibiotics (whole group)	Glycopeptide antibiotics (excl. Vancomycin)
	Vancomycin
Certain chemotherapy agents	Certain chemotherapy agents (excl. Taxanes)
	Taxanes
Contrast media (whole group)	Iodinated contrast media
	Other contrast media
Certain other analgesics	Paracetamol
	Metamizole (i.v.)
Biologicals	Biologicals with immunological target
	Biologicals without immunological target
Miscellaneous drugs	Miscellaneous drugs (excl. Protamine)
	Protamine
Serotonin syndrome	
Tricyclic/ tetracyclic antidepressants (whole group)	Tricyclic antidepressants (excl. Clomipramine/ Imipramine)
	Clomipramine and Imipramine
	Tetracyclic antidepressants
Agranulocytosis / Neutropenia	
Certain antivirals	Certain antivirals (excl. Ganciclovir and Valganciclovir)
	Ganciclovir and Valganciclovir
Certain immunosuppressants (excl. Biologicals)	Certain other immunosuppressants (excl. Biologicals, Azathioprine, Mycophenolate mofetil)
	Mycophenolic mofetil and Azathioprine
Acute kidney injury	
Polymyxins	Polymyxins (i.v)
Certain other antibiotics	Certain other antibiotics (excl. Vancomycin)
	Vancomycin
Certain antineoplastic agents	Certain other antineoplastic agents (excl. Methotrexate, Cisplatin, Ifosfamide)
	Methotrexate, Cisplatin, Ifosfamide

Initial drug class (round 1)	Adjusted drug class (round 2)
Certain antiviral agents	Certain antiretroviral therapy
	Certain other antivirals
mTor inhibitors/ Calcineurin inhibitors (whole group)	mTor inhibitors
	Calcineurin inhibitors
Rhabdomyolysis-inducing drugs	Excluded
Rhabdomyolysis	
Certain cytostatic drugs	Certain cytostatic drugs (excl. Trabectedin)
	Trabectedin
Delirium	
SSRI (whole group)	SSRI (excl. Paroxetine)
Anticonvulsants (excl. Phenobarbitals)	Anticonvulsants (excl. Phenobarbitals and Carbamazepine)
Antidiabetics	Excluded

Abbreviations: ASA: Acetylsalicylic acid; Excl.: Exclusive; mTOR: Mammalian target of rapamycin; NSAIDs: Non-steroidal anti-inflammatory drugs; SSRI: Selective serotonin reuptake inhibitors
